# Ubiquitin-like conjugation by bacterial cGAS enhances anti-phage defence

**DOI:** 10.1038/s41586-023-05862-7

**Published:** 2023-02-27

**Authors:** Justin M. Jenson, Tuo Li, Fenghe Du, Chee-Kwee Ea, Zhijian J. Chen

**Affiliations:** 1 Department of Molecular Biology, University of Texas Southwestern Medical Center, Dallas, TX USA; 2 Center for Inflammation Research, University of Texas Southwestern Medical Center, Dallas, TX USA; 3 Howard Hughes Medical Institute, University of Texas Southwestern Medical Center, Dallas, TX USA

**Keywords:** Innate immunity, Phage biology

## Abstract

cGAS is an evolutionarily conserved enzyme that has a pivotal role in immune defence against infection^1–3^. In vertebrate animals, cGAS is activated by DNA to produce cyclic GMP–AMP (cGAMP)^4,5^, which leads to the expression of antimicrobial genes^6,7^. In bacteria, cyclic dinucleotide (CDN)-based anti-phage signalling systems (CBASS) have been discovered^8–11^. These systems are composed of cGAS-like enzymes and various effector proteins that kill bacteria on phage infection, thereby stopping phage spread. Of the CBASS systems reported, approximately 39% contain *Cap2* and *Cap3*, which encode proteins with homology to ubiquitin conjugating (E1/E2) and deconjugating enzymes, respectively^8,12^. Although these proteins are required to prevent infection of some bacteriophages^8^, the mechanism by which the enzymatic activities exert an anti-phage effect is unknown. Here we show that Cap2 forms a thioester bond with the C-terminal glycine of cGAS and promotes conjugation of cGAS to target proteins in a process that resembles ubiquitin conjugation. The covalent conjugation of cGAS increases the production of cGAMP. Using a genetic screen, we found that the phage protein Vs.4 antagonized cGAS signalling by binding tightly to cGAMP (dissociation constant of approximately 30 nM) and sequestering it. A crystal structure of Vs.4 bound to cGAMP showed that Vs.4 formed a hexamer that was bound to three molecules of cGAMP. These results reveal a ubiquitin-like conjugation mechanism that regulates cGAS activity in bacteria and illustrates an arms race between bacteria and viruses through controlling CDN levels.

## Abstract (web summary)

Cap2 of the cyclic dinucleotide-based anti-phage immune system functions through a ubiquitin-like conjugation mechanism to regulate cGAS activity.

## Main

**Fig. 1:**
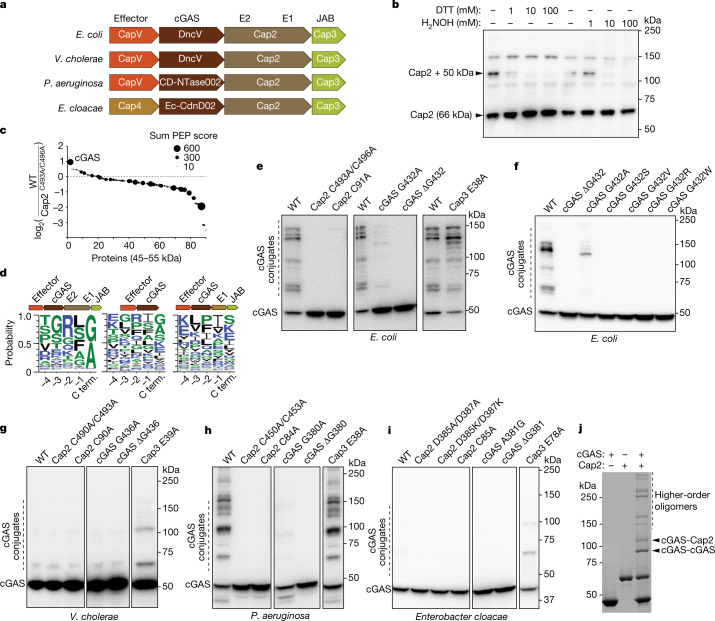
Cap2 catalyses covalent conjugation of cGAS. **a**, Domain organization of the CBASS operons from *E. coli*, *Vibrio cholerae*, *Pseudomonas aeruginosa* and *Enterobacter cloacae*. **b**, *E. coli* MG1655, which lacks CBASS, was transformed with the four-gene operon (from **a**) including an N-terminally Flag-tagged Cap2. Bacterial cell lysates were treated for 30 min with the indicated amount of DTT or hydroxylamine (H_2_NOH), subjected to non-reducing SDS–PAGE and immunoblotted with an anti-Flag antibody. **c**, Mass spectrometry analysis of 45–55 kDa proteins conjugated to Cap2 that migrated at about 110 kDa on an SDS–PAGE gel. The *y* axis indicates enrichment of the proteins in bacteria with wild-type CBASS compared to those harbouring the C493A/C496A mutations of Cap2. The sum PEP score is the sum of negative logarithms of the posterior error probabilities (PEP) and represents the credibility of the spectral matches. **d**, Sequence logo of alignment of the C-terminal (C term.) sequences of cGAS from previously identified CBASS operons^12^ with the indicated gene architecture: operons containing full-length Cap2 and Cap3 (left), minimal operons with no Cap2 or Cap3 (centre) and operons lacking the E2 domain in Cap2 (right). **e**–**i**, Immunoblotting of cGAS conjugates in bacterial lysates from cells harbouring the wild-tye (WT) four-gene CBASS operon with N-terminally Flag-tagged cGAS, or those with indicated mutations in the CBASS (**e**) and cGAS (**f**) genes from *E. coli*, *V. cholerae* (**g**), *P. aeruginosa* (**h**) and *Enterobacter cloacae* (**i**). Bacteria cell lysates were subjected to SDS–PAGE in the presence of β-mercaptoethanol, followed by immunoblotting with an anti-Flag antibody. **j**, Purified recombinant cGAS and Cap2 proteins were incubated in the presence of ATP for 30 min at room temperature, as indicated. Following the reaction, proteins were separated by SDS–PAGE and stained with Coomassie blue. All data are representative of at least two independent experiments. For gel source data, see Supplementary Fig. 1. Source data

Bioinformatic analysis has identified more than 2,000 cyclic dinucleotide (CDN)-based anti-phage signalling systems (CBASS) operons that contain two ancillary genes, *Cap2* and *Cap3*, which encode proteins with ubiquitin conjugating and deconjugating enzyme domains, respectively (Fig. 1a)^8,12^. *Cap2* encodes an E1 ubiquitin activating enzyme domain and an E2 ubiquitin carrier domain. Cap3 contains a JAB deubiquitinase domain. No E3-like ubiquitin ligase or ubiquitin-like protein has been found to function in bacterial immune defence systems^13^. In addition to *Cap2*, *Cap3* and *cGAS*, CBASS operons contain genes encoding effector proteins such as CapV, a phospholipase that is activated by cGAMP to degrade the bacterial membrane and kill the bacteria to prevent phage propagation in the bacterial community^11^ (Fig. 1a).

## Cap2 catalyses conjugation of cGAS

Because the E1 and E2 domains of Cap2 are expected to form thioester bonds with their targets as a key part of their reaction mechanism, we searched for covalent adducts in the lysates of bacteria containing the CBASS operon from *Escherichia coli* TW11681, to find evidence of conjugation to a ubiquitin-like protein. The CBASS operon was transferred to *E. coli* MG1655, which lacks endogenous CBASS. Western blots of Flag-tagged Cap2 revealed the presence of a covalent adduct that was sensitive to dithiothreitol (DTT) or hydroxylamine, consistent with the formation of a thioester bond (Fig. 1b). The observed mass shift of approximately 50 kDa was much larger than the known bacterial ubiquitin-like proteins in *E. coli*, including ThiS (7.3 kDa) and MoaD (8.8 kDa). To identify the protein present in this complex, we searched for 45–55 kDa proteins that migrated at about 110 kDa on an SDS–PAGE gel using mass spectroscopy. As a control, we used bacteria with point mutations in the cysteine residues (C493A/C496A) predicted to be the E1 active site of Cap2. *E. coli* cGAS (also known as DncV^14^) was enriched in the sample with wild-type Cap2 (Fig. 1c), suggesting that it may be the target of Cap2. This was unexpected because cGAS lacks the highly conserved ubiquitin β-grasp protein fold and does not contain two glycine residues at its C terminus, which are required by most ubiquitin-like proteins to form covalent intermediates with E1/E2 enzymes (although there are notable exceptions to this rule, such as the Atg8/LC3 family, which has a single C-terminal glycine). Alignment of the C termini of 1,583 cGAS proteins^12^ from operons that contain full-length Cap2 and Cap3 showed the C-terminal residue to be highly conserved, as either glycine or alanine (Fig. 1d). By contrast, the penultimate residues vary. We found no conservation in the C-terminal residues of cGAS in CBASS operons with other architectures, including those that lack Cap2 and Cap3, and those lacking an E2 domain (Fig. 1d).

**Extended Data Fig. 1:**
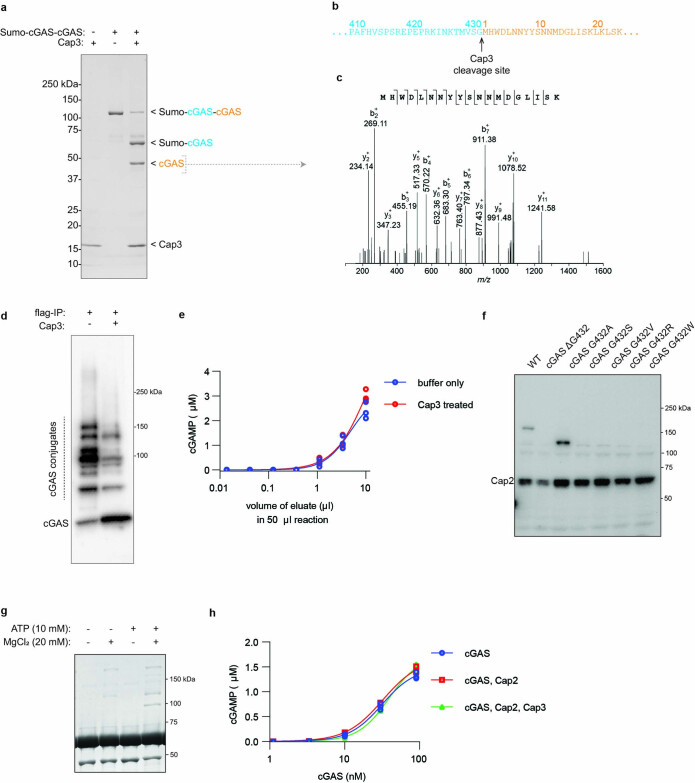
Conjugation of cGAS and deconjugation by Cap3. **a**, Cap3 cleaves at c-terminus of cGAS. Recombinant Cap3 was incubated with Sumo-cGAS-cGAS for 120 min at 37 °C, separated with SDS-PAGE, and stained with Coomassie blue. Data is representative of 3 independent experiments. The indicated bands were analyzed by LC-MS/MS. **b**, The Cap3 cut site identified by MS. **c**, A tandem mass spectrum of a peptide fragment from the Cap3 cleavage product showing the N-terminus of cGAS after cleavage from Sumo-cGAS. **d**, Flag-tagged cGAS proteins were immunopurified from cells harboring CBASS with inactivated Cap3. The isolated proteins were treated with Cap3 for 120 min at 37 °C followed by SDS-PAGE and immunostaining with an anti-Flag antibody. Data is representative of 2 independent experiments. **e**, cGAMP production of Cap3 treated and untreated isolates from d was measured using THP1-Lucia ISG cells (methods). Scatter plot represents mean ± SD of n = 3 technical replicates with individual points overlaid and is representative of two independent experiments. **f**, Immunoblot of cGAS thioester in bacterial lysates from cells harboring a CBASS operon with N-terminally Flag-tagged Cap2 and the indicated mutations. Cell lysates were subjected to non-reducing SDS-PAGE and immunoblotted with an anti-Flag antibody. The data are representative of two independent experiments. **g**, Recombinant cGAS and Cap2 proteins were incubated for 30 min at room temperature, separated by SDS-PAGE, and stained with Coomassie blue. Data are representative of two independent experiments. **h**, Enzymatic activity of recombinant cGAS in the presence of Cap2 and Cap3 was measured by incubating with ATP to allow conjugate formation before supplementing with ATP, GTP, and MgCl_2_ then incubating for 30 min at 37 °C. cGAMP generated in the reaction was measured in THP1-Lucia ISG cells. Scatter plot represents mean ± SD of n = 3 technical replicates with individual points overlaid and is representative of two independent experiments. For gel source data, see Supplementary Fig. 1. Source data

To test whether cGAS is the target of Cap2, we performed immunoblotting of bacterial lysates containing Flag-tagged cGAS from *E. coli* TW11681 and observed extensive cGAS conjugation (Fig. 1e). This conjugation required the activity of both the E1 and E2 domains of Cap2 (Fig. 1e). We also observed that mutations of the C terminus of cGAS had pronounced effects on its conjugation. Deletion of the conserved C-terminal glycine abolished cGAS conjugation (Fig. 1e), as did mutating this residue to serine, valine, arginine or tryptophan (Fig. 1f). Mutation of the C-terminal glycine to alanine reduced, but did not abolish, cGAS conjugation (Fig. 1f), which is consistent with the conservation of alanine in the C termini of some bacterial cGAS (Fig. 1d). Additionally, we found that mutation of a putative catalytic residue of Cap3 (E38A), which abrogates its isopeptidase activity, increased cGAS conjugation, suggesting that Cap3 functions as a cGAS isopeptidase (Fig. 1e). To directly test whether Cap3 is an enzyme that cleaves cGAS from its conjugates, we generated a protein in which Sumo is fused to two molecules of cGAS linked in tandem (Sumo–cGAS–cGAS; Extended Data Fig. 1a). Treatment of this fusion protein with recombinant Cap3 protein revealed that Cap3 cleaved specifically at the C terminus of the first cGAS (Extended Data Fig. 1a–c). We also isolated cGAS conjugates from *E. coli* by immunoprecipitation and found that cGAS could be cleaved from these conjugates by the Cap3 protein in vitro (Extended Data Fig. 1d). However, we found that the deconjugation activity of Cap3 had negligible effect on cGAMP production by the cGAS conjugates (Extended Data Fig. 1e).

To determine whether cGAS conjugation is a widespread mechanism common to diverse bacteria, we cloned CBASS operons from *Vibrio cholerae*, *Pseudomonas aeruginosa* and *Enterobacter cloacae*, all of which contain *Cap2* and *Cap3* in addition to *cGAS* and an effector gene (*CapV* or *Cap4*), and tested their activities in *E. coli* MG1655. Although these operons differed in the identities of the cell-death effectors and cGAS-like enzymes that produced distinct cyclic oligonucleotides, the cGAS proteins all contained the conserved C-terminal glycine (*V. cholerae* and *P. aeruginosa*) or alanine (*E. cloacae*). All three of these systems were capable of cGAS conjugation (Fig 1g–i). The cGAS protein (CD-NTase002) from *P. aeruginosa* formed extensive conjugates (Fig. 1h). This conjugation was dependent on activity of both E1 and E2 domains of Cap2 and required a glycine at the C terminus of cGAS (Fig. 1h). For the operons from *V. cholerae* and *E. cloacae*, cGAS conjugation was most apparent when the Cap3 activity was disrupted (Fig. 1g,i), which provides additional evidence of its isopeptidase function.

Consistent with ubiquitin-like conjugation by cGAS, we found that the C-terminal glycine of cGAS is important for forming a thioester with Cap2. Removing this residue abrogated the formation of the Cap2–cGAS thioester, as did mutating this residue to serine, valine, arginine or tryptophan (Extended Data Fig. 1f). By contrast, mutating this residue to alanine increased the amount of Cap2–cGAS thioester. One possible explanation for this increase is that this mutation may hinder the transfer of cGAS from the thioester to its targets, as shown in Fig. 1e,f, leading to an accumulation of Cap2–cGAS thioester intermediates. To further confirm that cGAS is the target of Cap2, we purified recombinant cGAS and Cap2 proteins and tested their activity in vitro. cGAS conjugates formed in the presence of Cap2 (Fig. 1j), and this reaction was ATP dependent (Extended Data Fig. 1g), although the conjugation that was formed in vitro did not affect cGAS activity (Extended Data Fig. 1h).

## cGAS conjugation reduces phage infection

**Fig. 2:**
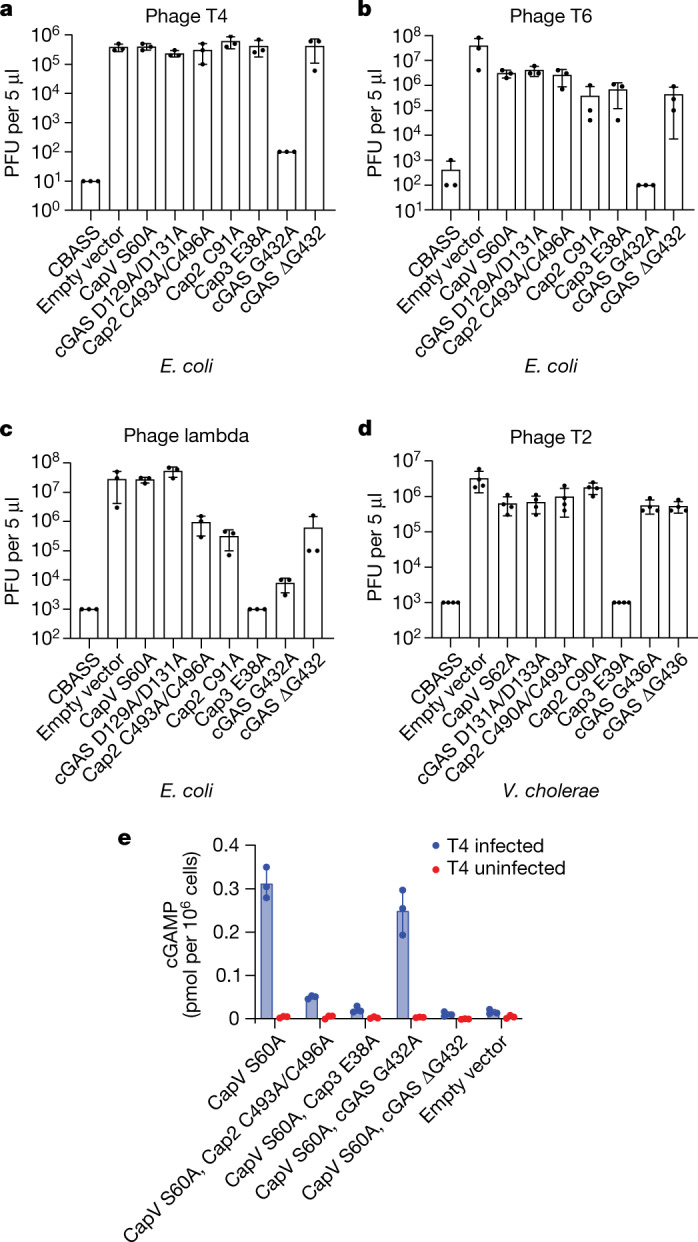
cGAS conjugation enhances cGAMP production and anti-phage immunity in vivo. **a**–**c**, Viral titre of phage T4 (**a**), T6 (**b**) and lambda (**c**) after infection of bacterial strains with the wild-type operon from *E. coli* (CBASS), no operon (empty vector) or the indicated point mutations. Data are mean ± s.d. of *n* = 3 independent experiments with individual points overlaid. **d**, Viral titre of phage T2 after infection in bacterial strains harbouring the wild-type *V. cholerae* CBASS operon, no operon (empty vector) or the indicated point mutations. Data are mean ± s.d. of *n* = 4 independent experiments with individual points overlaid. **e**, *E. coli* harbouring the indicated CBASS operon was infected by the phage T4 at a multiplicity of infection of approximately 10. CapV in the CBASS operon was inactivated by a mutation in the active site (S60A) to avoid cell death from CBASS signalling. Samples from each bacterial culture were collected 60 min after infection, snap frozen and lysed by heating. Clarified lysates were incubated with THP1 Lucia ISG cells, which express a luciferase (Lucia) reporter gene under the control of an IRF-inducible promoter. Luminescence signal was measured and converted to cGAMP concentrations using a cGAMP standard curve. Data are mean ± s.d. of *n* = 3 technical replicates and is representative of two independent experiments. Source data

**Extended Data Fig. 2:**
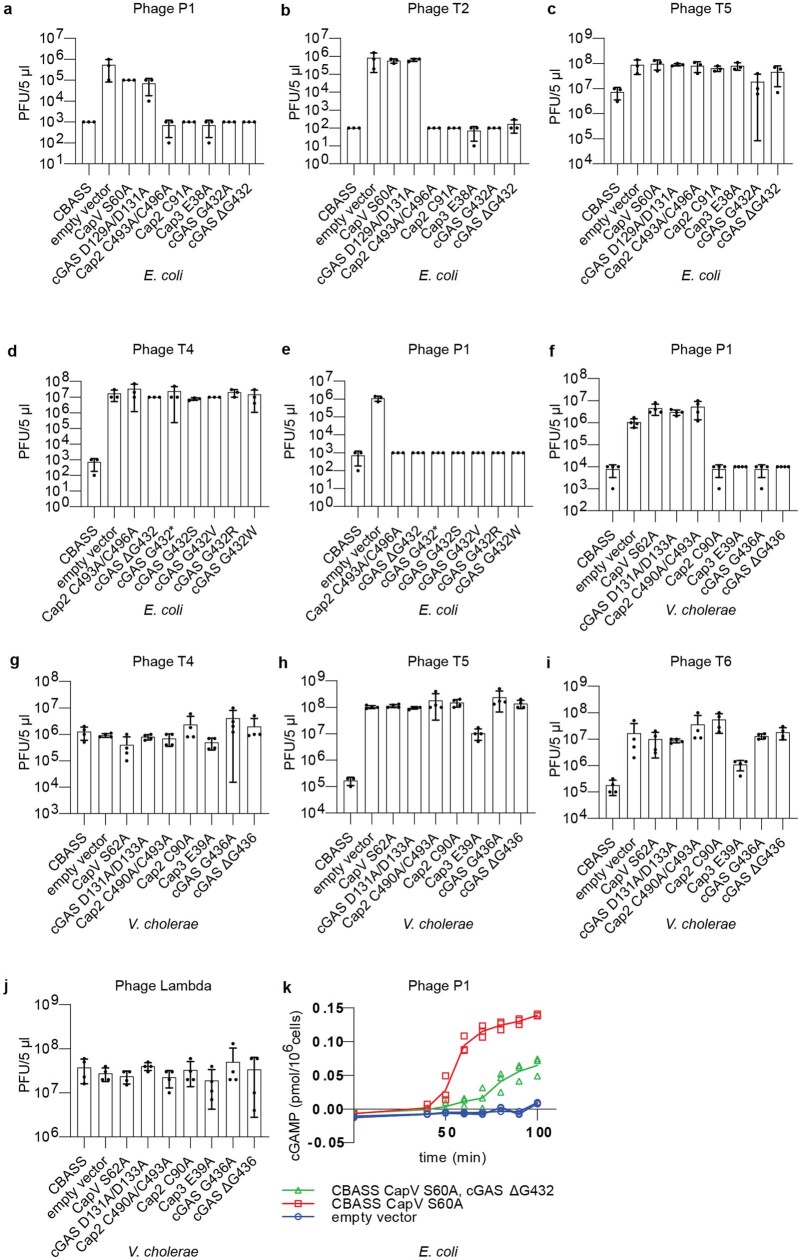
Viral titers of bacteriophages in bacteria harboring CBASS operons with point mutations in the indicated genes. **a**–**c**, *E. coli* MG1655 was transformed with the wild-type operon from *E. coli* TW11681 (CBASS) and the operon containing the indicated mutations. Empty vector indicates no CBASS operon. The strains were infected with Phage P1 (a), Phage T2 (b), or Phage T5 (c) and the plague forming units (PFU) were measured. Bar graph represents mean ± SD of n = 3 independent experiments for each bacterial strain. **d**–**e**, Viral titers of phage T4 (d) and P1 (e) after infection in bacterial strains harboring the wild-type CBASS operon from *E. coli* TW11681, no operon (empty vector), or the indicated point mutations. Bar graph represents average values ± SD of n = 3 independent experiments with individual points overlaid. **f**–**j**, *E. coli* MG1655 was transformed with the wild-type operon from *V. cholerae* (CBASS) with Flag-tagged cGAS and the indicated mutations. Empty vector indicates no CBASS operon. The strains were infected with  Phage P1 (f), Phage T4 (g), Phage T5 (h), Phage T6 (i), or Phage Lambda (j) and the plague forming units (PFU) were measured. Bar graph represents mean ± SD of n = 4 independent experiments for each bacterial strain. **k**, *E. coli* harboring the indicated CBASS operon was infected by the phage P1 at a multiplicity of infection of 2. CapV in the CBASS operon was inactivated by a mutation in the active site (S60A) to inhibit cell death from CBASS signaling. Samples from each bacterial culture were collected at 10-min intervals starting at 40 min after infection, snap frozen, and lysed by heating and sonication. Clarified lysates were incubated with purified CapV in the presence of resorufin butyrate, a fluorogenic phospholipase substrate, to measure the bioactivity of cGAMP produced by the bacteria. Scatter plot represents mean ± SD of n = 3 technical replicates with individual points overlaid and is representative of two independent experiments. Source data

The catalytic activity of Cap2 is required for CBASS-mediated immunity against some phages^8^. Thus, we tested whether conjugation by cGAS is important for the antiviral effect of Cap2. As previously reported, mutations of the active site cysteine residues in the E1 or E2 domains of Cap2 increased the propagation of T4, T6 and lambda phages by more than two orders of magnitude (Fig. 2a–c), but did not affect the titre of P1, T2 and T5 phages (Extended Data Fig. 2a–c). For T4 and T6 phages, mutating Cap2 disrupts anti-phage defence similarly to the mutations that inactivate the catalytic activities of cGAS and CapV (Fig. 2a,b). For phage lambda, mutating Cap2 partially disrupted phage defence, leading to a more than 100-fold increase in susceptibility to phage infection, whereas inactivating cGAS or CapV increased the viral titre by more than four orders of magnitude (Fig. 2c). Importantly, inhibiting cGAS conjugation by deleting the C-terminal glycine phenocopied the effects of inactivating Cap2 (Fig. 2a–c), as did mutating the C-terminal glycine to serine, valine, arginine or tryptophan (Extended Data Fig. 2d,e). Mutating the C-terminal glycine of cGAS to alanine, which reduced but did not completely inhibit cGAS conjugation, partially reduced the protection against the T4 and lambda phages provided by CBASS (Fig. 2a,c), but did not compromise the protective effect of CBASS against the T6 phage (Fig. 2b). Similar to the *E. coli* system, we found that cGAS conjugation was required by the *V. cholerae* CBASS system to protect against some phages (T2, T5 and T6) but not others (P1, T4 and lambda; Fig. 2d and Extended Data Fig. 2f–j). These results strongly suggest that the ubiquitin-like conjugation through the C terminus of cGAS is important for anti-phage immune defence and explains why the mutations of E1/E2 active sites of Cap2 abrogated the CBASS function.

## cGAS conjugation enhances cGAMP production

To elucidate how cGAS conjugation expands the range of phages that CBASS protects against, we tested whether cGAS conjugation stimulates its activity by measuring cGAMP production in phage-infected bacteria with and without conjugated cGAS (Fig. 2e). In these experiments, we used an *E. coli* strain with inactive CapV to prevent abortive cell death so that we could measure cGAMP production in phage-infected cells. T4 phage infection led to induction of cGAMP in a manner that depended on the activity of Cap2 and Cap3 (Fig. 2e). Deletion of the C-terminal glycine, but not the glycine to alanine substitution, abolished cGAMP production, consistent with the results of the anti-phage defence assay (Fig. 2a). Similar to T4 phage, P1 phage, which requires cGAS but not Cap2 to inhibit phage propagation (Extended Data Fig. 2a), induced approximately tenfold more cGAMP in cells expressing WT cGAS than in cells expressing cGAS lacking the C-terminal glycine (Extended Data Fig. 2k). It is possible that the low level of cGAMP produced by cGAS in cells that do not have the conjugation system is sufficient to protect against infection by P1, but not by T4, phage. These data suggest that Cap2-mediated conjugation enhances cGAS activity.

**Extended Data Fig. 3:**
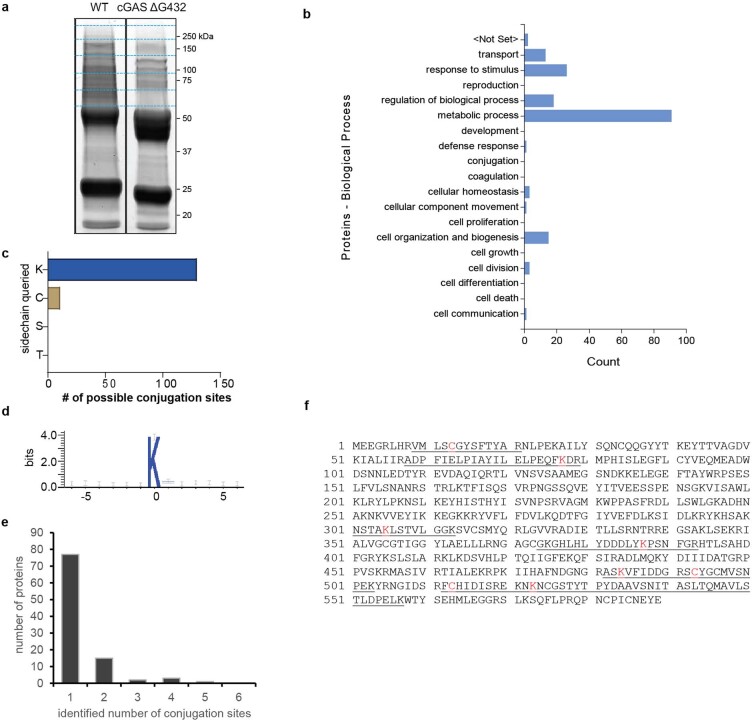
Identification of cGAS conjugation sites. **a**, Flag-tagged cGAS and cGAS ΔG432 proteins were immunopurified from *E. coli* cells harboring the CBASS operons encoding these proteins. Isolated proteins were separated on SDS-PAGE and stained by Coomassie blue. Indicated protein bands were cut, alkylated, digested with trypsin, and analyzed by shot-gun LS-MS/MS analysis. Data is representative of three independent experiments: two with uninfected cells and one with cells infected with phage T4. **b**, Biological processes associated with proteins found to be conjugated by cGAS. **c**, The numbers of conjugation sites identified for lysine, cysteine, serine, and threonine is shown. **d**, Sequence alignment of the 130 cGAS conjugation sites surrounding the target lysine. **e**, Histogram of the number of conjugation sites per cGAS conjugated target. **f**, The protein sequence of Cap2. The cGAS conjugation sites are indicated in red and the peptides in which conjugation sites were identified by mass spectrometry are underlined. For gel source data, see Supplementary Fig. 1. Source data

Next, we attempted to identify the targets of cGAS conjugation by immunoprecipitating Flag-tagged cGAS from both uninfected and infected *E. coli*, digesting the enriched proteins with trypsin and then using mass spectrometry to identify peptides that contain the C-terminal remnant of cGAS (Thr-Met-Val-Ser-Gly; Extended Data Fig. 3a). We searched for mass shifts on peptides with several nucleophilic amino acid side chains and found that, of the side chains searched, lysine was the predominant cGAS conjugation target, with 130 target sites identified (Extended Data Fig. 3c). In this configuration, the C terminus of cGAS forms an isopeptide bond with the ε-amine of a lysine, which is similar to ubiquitination in eukaryotes. Other than lysine, ten cysteines were found attached to the cGAS C-terminal remnant as thioesters, three of which were found on Cap2 at positions C13, C493 and C513 (Extended Data Fig. 3f). On the basis of homology with ThiF^15^, cysteine 513 would be predicted to be a catalytic cysteine in the E1 domain and to form a thioester bond with cGAS. No conjugation scars were found on serine or threonine. How lysines are selected for conjugation is unclear, as there is no obvious consensus in the sequences flanking the attachment site (Extended Data Fig. 3d). We found that most cGAS-conjugated proteins were only modified on a single site (Extended Data Fig. 3e), although a few were more heavily modified. The protein with the most unique conjugation sites detected was Cap2, which was found to be modified by cGAS at five different lysine residues (Extended Data Fig. 3f). This may explain some DTT-insensitive ‘background’ Cap2-conjugated bands that were observed in the whole lysate in Fig. 1b. cGAS was predominantly conjugated to proteins involved in metabolism (Extended Data Fig. 3b and Supplementary Table 1). We found that during phage T4 infection, cGAS was conjugated to at least two phage proteins. One of these phage proteins is fibritin, a protein involved in tail fibre assembly, which was found to have cGAS linked to lysine 100. The other phage protein we identified is an uncharacterized 9.5 kDa protein, DexA.2, which had cGAS modification detected on lysine 5 (Supplementary Table 2). Together, these biochemical and proteomic data suggest that Cap2 can covalently conjugate the cGAS C terminus to the ε-amino group of a lysine on target proteins from both *E. coli* and T4 phage, but we cannot rule out the possibility that cGAS might also be conjugated to non-protein targets^16,17^.

## Vs.4 sequesters cGAMP to counter CBASS

**Fig. 3:**
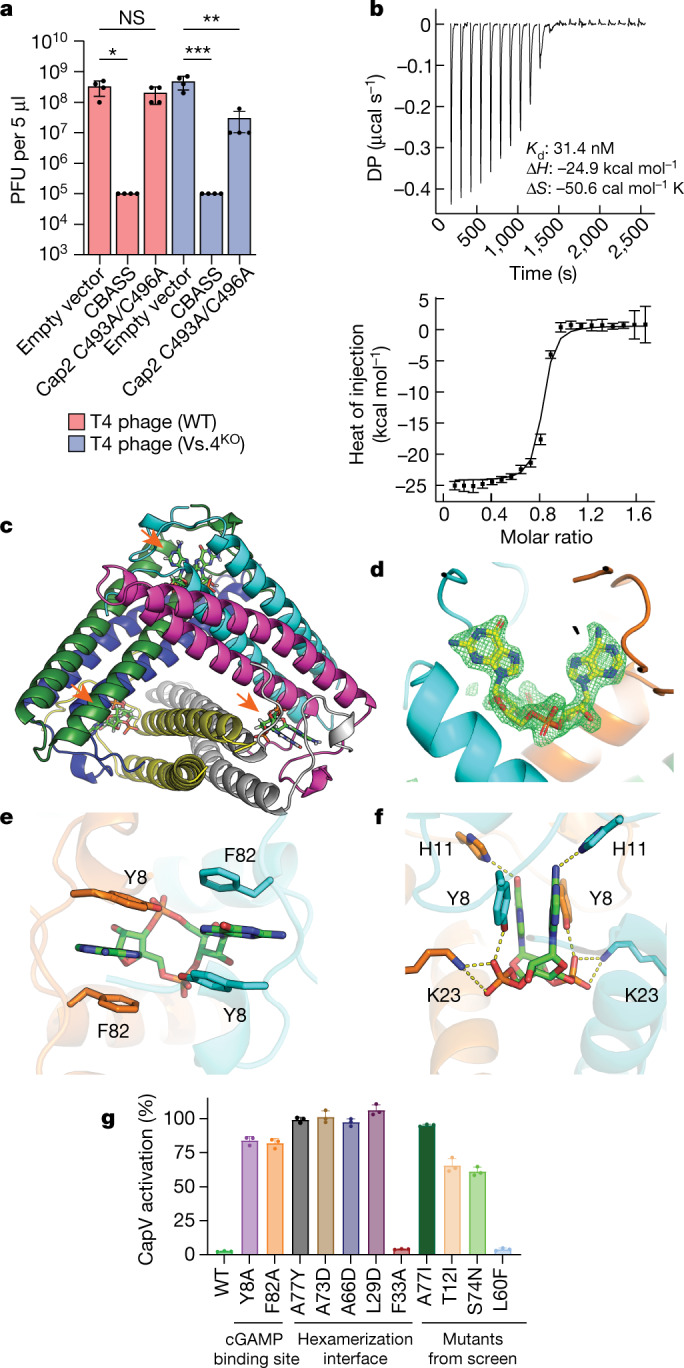
Vs.4 tightly binds and sequesters cGAMP. **a**, Viral titre of WT and Vs.4 knockout T4 phage in strains of *E. coli* MG1655 containing the WT or Cap2 C493A/C496A CBASS or empty vector. Bar graph represents average values ±s.e.m. of *n* = 4 independent experiments with individual points overlaid. NS, not significant, *P < 0.05 (*P* = 0.0178), **P < 0.005 (*P* = 0.0010) and ***P < 0.0005 (*P* = 0.0005) by one-way analysis of variance test. **b**, Representative example of a raw titration trace of differential power (DP) versus time (top) and integrated data (bottom, data points depict integrated heat of injection and error bars depict the weighted root mean squared deviation of the difference between predicted and measured values) of an ITC experiment in which cGAMP was titrated into a solution of Vs.4. The global best fit of three titrations indicated a dissociation constant (*K*_d_) of 31.4 nM with a 68% confidence interval of 18–47.5 nM. **c**, X-ray crystal structure of hexameric Vs.4 (cartoon representation, one colour per monomer) bound to three molecules of cGAMP (green stick representation indicated by orange arrows). **d**, Composite omit map calculated with the cGAMP ligand omitted. Simulated annealing was used to remove any memory of the ligand. The difference density map (green) is contoured at 3.5*σ* with cGAMP (yellow) superimposed in the binding pocket that is formed between two monomers (cyan and orange). **e**, Detailed view of the residues forming π–π interactions with cGAMP. **f**, Detailed view of hydrogen bonds and salt bridges formed with cGAMP in the Vs.4 binding pocket. **g**, CapV was incubated with the indicated Vs.4 mutant (10 µM), cGAMP (1 µM) and resorufin butyrate (a fluorogenic phospholipase substrate) to measure the enzymatic activity of CapV. Bar graph represents average values ±s.d. of *n* = 3 technical replicates with individual points overlaid and is representative of two independent experiments. Source data

**Extended Data Fig. 4:**
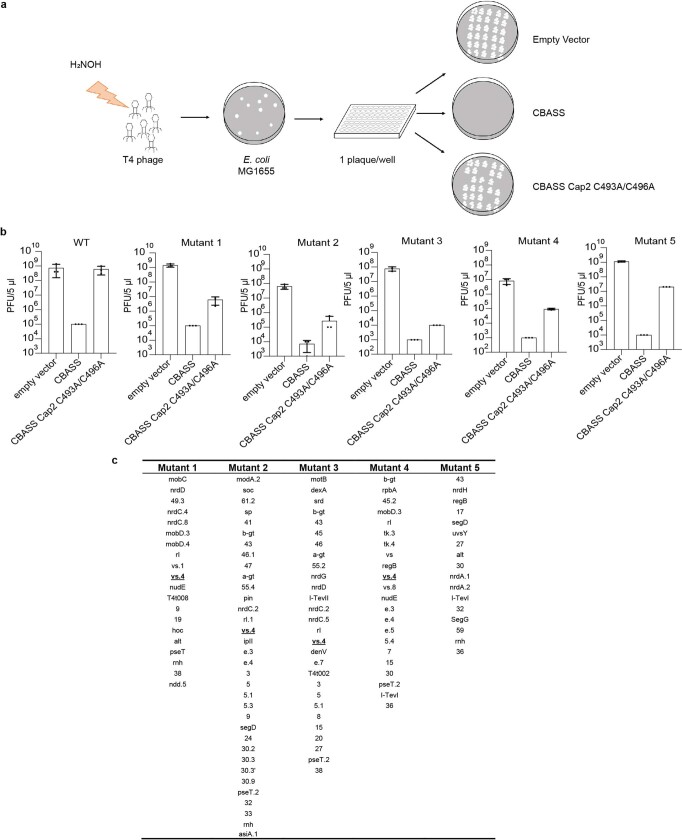
Screen for loss of function mutants of T4 phage. **a**, T4 phage was mutagenized with hydroxylamine (NH_2_OH) to produce a library, mixed with wild-type *E. coli* MG1655 and plated (Methods). Individual plaques (mutants) from this library were picked and transferred to wells of 96-well plates. These phage-containing solutions were pipetted onto the surface of top agar that had been inoculated with *E. coli* MG1655 harboring no CBASS (empty vector), wild-type CBASS (CBASS), or CBASS with inactivating Cap2 mutations (C493A/C496A). Mutants that formed plaques only on the empty vector plates were selected for further validation. **b**, efficiency of plating of wild-type T4 phage (WT) and the five mutant T4 phages (Mutants1–5) with more than 50-fold reduction in infectivity in bacteria containing the Cap2 C493A/C496A mutations. Bar plots represent mean ± SD of n = 3 independent experiments. **c**, table of genes found with non-synonymous mutations found in T4 phage mutants with increased sensitivity to CBASS defective in cGAS conjugation. Source data

**Extended Data Fig. 5:**
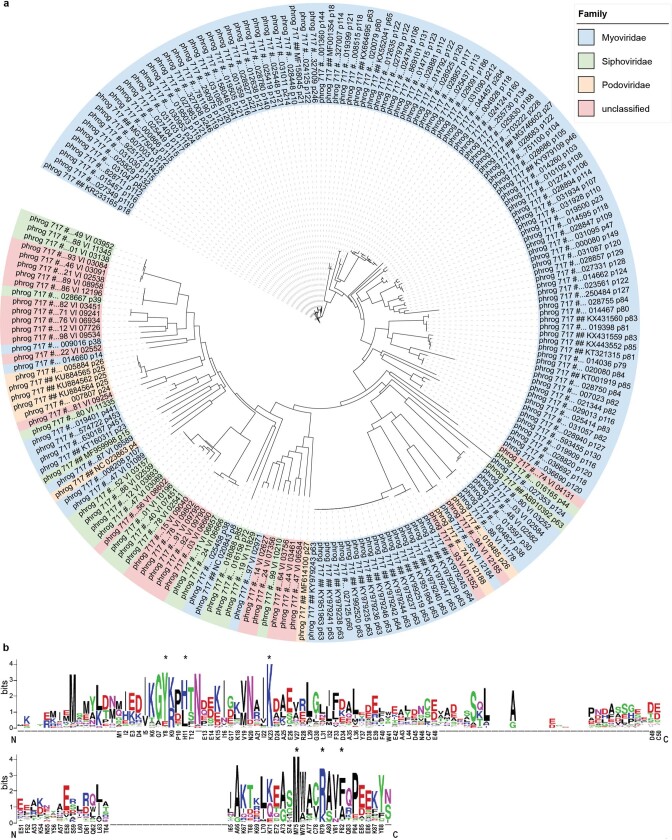
Proteins with homology to Vs.4 are found in diverse phages. **a**, Phylogenetic tree of 198 proteins with predicted homology to Vs.4 identified in Prokaryotic Virus Remote Homologous Groups database (see Methods). **b**, Vs.4 homologs were aligned with clustal omega and the multiple sequence alignment is shown. The sequence of Vs.4 from T4 phage is displayed on the x-axis. Insertions in the alignment are denoted with dashes. Residues in the cGAMP binding site are indicated with an *.

To understand why cGAS conjugation is required for CBASS to protect against T4 phage, we performed a forward genetic screen. The goal of this screen was to identify potential cGAS conjugation targets in phages that could provide mechanistic insight into how cGAS conjugation enhances cGAMP production and inhibits phage propagation. We reasoned that if the phage encodes a conjugation target that enhances cGAS activity, it may be possible to find mutants that can propagate in the presence of the CBASS system. Alternatively, if the phage encodes a cGAS or CapV inhibitor that is antagonized by conjugation, we may find phage mutants that are sensitive to CBASS that is defective in conjugation. To test both possibilities, we mutagenized T4 phage with hydroxylamine, and screened for phage growth on bacteria containing a WT CBASS operon, those containing a CBASS operon with a catalytically inactive Cap2 (C493A/C496A) enzyme and those with a vector-only control (Extended Data Fig. 4a). Although we have not yet identified a cGAS conjugation target in phages from this screen, we isolated five T4 mutant phages that had a more than 50-fold decrease in their ability to infect the bacteria with catalytically dead Cap2 relative to wild type phage (Extended Data Fig. 4b). These results suggested that cGAS conjugation may help overcome cGAS or CapV inhibition by a T4 phage encoded factor. Genomic sequencing revealed candidate genes possessing mutations that might underlie the observed phenotypes (Extended Data Fig. 4c). The most frequently mutated gene in these mutants encodes Vs.4, a protein of unknown function. Vs.4 mutations were found in four out of the five mutants. Each of these mutants contained a different mutation: A77I, T12I, S74N and L60F. Knocking out Vs.4 in T4 phage revealed that the titre of this mutant phage in Cap2 mutant cells was approximately 50-fold less than the wild-type phage, whereas the abilities of these phages to infect cells lacking the CBASS system were similar (Fig. 3a). Bioinformatic analyses identified 198 homologues of Vs.4 in diverse phages (Extended Data Fig. 5a,b). These results suggest that Vs.4 is a conserved phage protein that may play a role in antagonizing CBASS activation.

**Extended Data Fig. 6:**
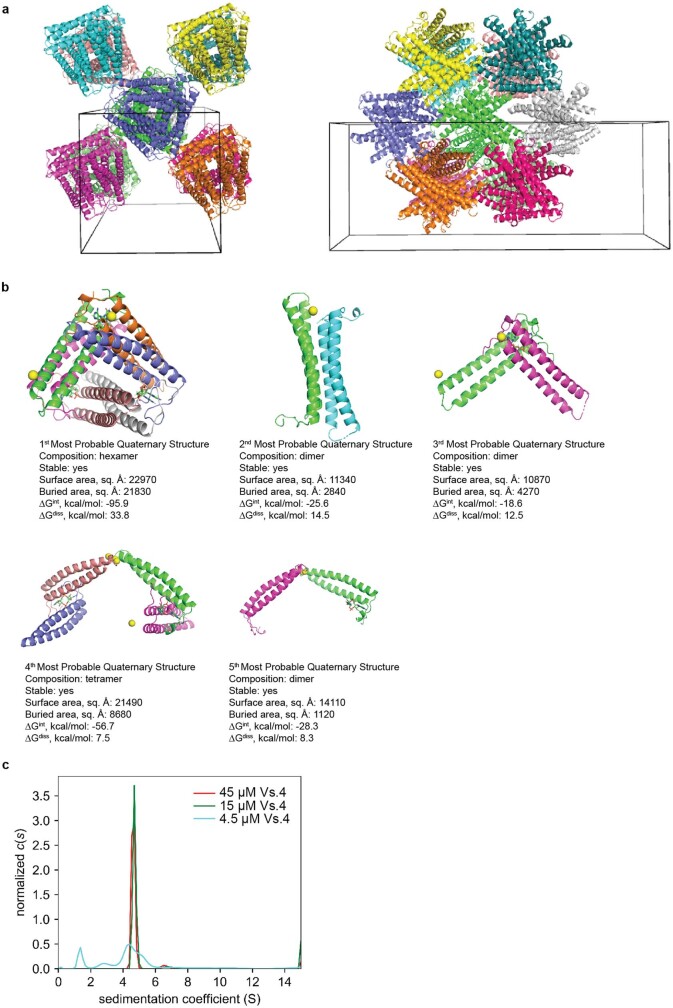
Quaternary structure assignment of Vs.4. **a**, schematic representation of crystal packing and unit cell of Vs.4. Monomers of Vs.4 cluster in hexamers in the lattice. To illustrate hexamer clusters, each hexameric complex of Vs.4 is highlighted in a unique color. **b**, PISA was used to analyze the Vs.4 structure for possible higher-order assemblies of Vs.4. In total, 5 assemblies were predicted. The top prediction (top left) is the only hexameric assembly that was predicted to be stable (see Methods). **c**, analytical ultracentricugation of Vs.4 bound to cGAMP. Vs.4 forms a hexaxamer that is stable on the time scale of the sedimentation experiment at 45 μM and 15 μM with a sedimentation coefficient of 4.64 and a molecular weight >50.3 kDa (61.8 kDa expected for hexamer). Source data

**Extended Data Fig. 7:**
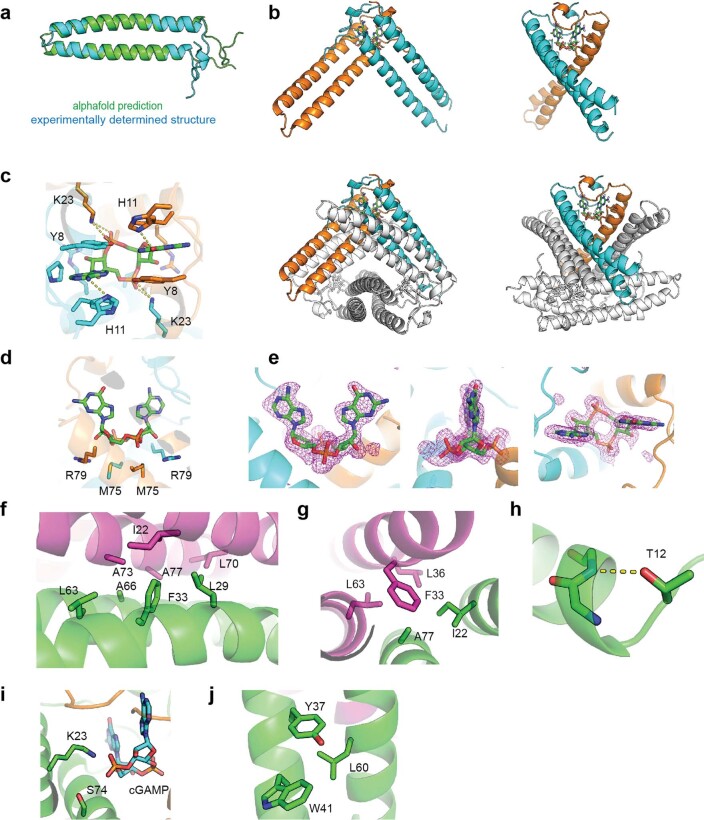
Structural analysis of the Vs.4:cGAMP complex. **a**, Alignment of the alphafold predicted structure of the Vs.4 monomer that was used for molecular replacement and the experimentally determined structure of Vs.4 bound to cGAMP. **b**, A cartoon representation of a single cGAMP binding pocket formed by Vs.4 dimers with one chain colored orange and the other cyan. cGAMP is bound between these monomers (green sticks). Other monomers are colored white to display the dimer in the context of the larger hexameric structure. **c**, Detail of the hydrogen-bonds formed with cGAMP in the binding pocket of Vs.4. **d**, Additional interactions between Vs.4 and cGAMP in the binding pocket. **e**, Polder map for the cGAMP ligand, which was calculated with cGAMP with 5Å radius omitted. The difference density map is contoured 3.0σ with cGAMP superimposed. **f**, Hexamer interface with key residues highlighted. **g**–**j**, Residues found mutated in forward genetic screen shown in Extended Data Fig. 4a: A77I sterically clashes with the heximerization interface (g); T12I removes a stabilizing helix cap (h); S74N interferes with interactions with cGAMP (i); and the L60F may contribute to steric clashes (j).

Considering that cGAS conjugation increased cGAMP production during infection, we examined whether Vs.4 might antagonize cGAS signalling by binding directly to cGAMP. Isothermal calorimetry (ITC) experiments showed that purified Vs.4 protein bound to cGAMP with a *K*_d_ of approximately 30 nM (Fig. 3b). To further investigate this interaction, we determined the crystal structure of Vs.4 bound to cGAMP at a resolution of 2.0 Å. This structure revealed that Vs.4 formed a hexameric complex that bound to three cGAMP molecules (Fig. 3c,d, Extended Data Fig. 6a,b and Extended Data Table 1). Analytical ultracentrifugation confirmed that Vs.4 formed a hexamer in solution (Extended Data Fig. 6c). Within the hexamer, each cGAMP binding site is composed of two Vs.4 monomers (Extended Data Fig. 7a,b), which make extensive π–π interactions between the purine bases of cGAMP and Y8/F82 of Vs.4 (Fig. 3e). Extensive hydrogen bonds and salt bridges were observed between cGAMP and Y8, H11, K23 and R79 of Vs.4 (Fig. 3f and Extended Data Fig. 7c–e). To validate the experimental structure and to test the hypothesis that Vs.4 could bind cGAMP and sequester it from the effector protein CapV, we used an in vitro CapV activity assay, which showed that recombinant Vs.4 inhibited cGAMP-mediated activation of CapV (Fig. 3g). Importantly, we found that Vs.4 mutants harbouring mutations to either the cGAMP binding interface (Y8 and F82) or the hexamerization interfaces (L29, A66, A73 and A77; Extended Data Fig. 7f) failed to inhibit CapV (Fig. 3g). Of the Vs.4 mutations identified from our forward genetic screen, A77I (which sterically clashes with the hexamerization interface), T12I (which removes a stabilizing helix cap) and S74N (which interferes with interactions with cGAMP) mutations were found to disrupt the ability of Vs.4 to inhibit CapV (Fig. 3g and Extended Data Fig. 7g–i). The L60F mutation did not abrogate the ability of Vs.4 to inhibit CapV activation by cGAMP (Fig. 3g). It is unclear why L60F was identified in our screen, although the structure suggests that this mutation may introduce steric clashes (Extended Data Fig. 7j). We do not know what effect this mutation has in vivo because we were unable to produce a phage with this point mutation, despite repeated attempts.

**Extended Data Fig. 8:**
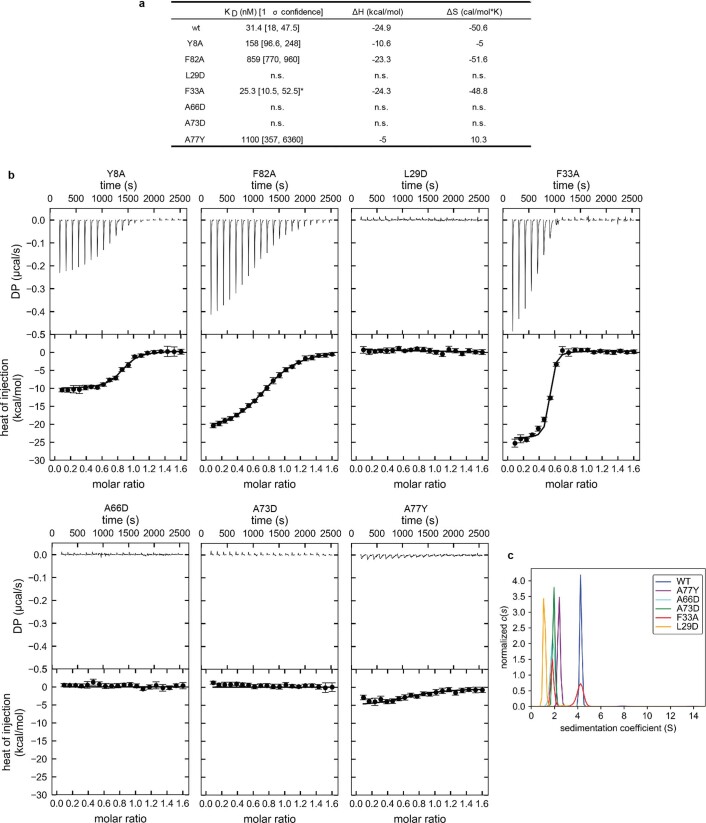
Mutational analysis of the Vs.4:cGAMP complex. **a**, Isothermal calorimetry (ITC) data from titrating cGAMP into a solution of the indicated Vs.4 mutant. Each experiment was performed at least twice and global analysis was used to determine K_D_, ΔH, and ΔS. Analysis of the F33A mutant indicated high incompetent fraction (0.496), as indicated with an *. Cases where no binding was observed are denoted as having no signal (n.s). **b**, Representative examples of raw titration traces (top) and integrated data (bottom, data points depict integrated heat of injection and error bars depict the weighted rmsd of the difference between predicted and measured values) of two independent ITC experiments reported in a. Titration data for WT Vs.4 is shown in Fig. 3b. **c**, Analytical ultracentrifugation of Vs.4 mutants. Vs.4 (WT) forms a hexamer that is stable on the time scale of the sedimentation experiment at 15 μM with a sedimentation coefficient of 4.3 and an estimated MW of 59 kDa (61.8 kDa expected). Mutations to the hexamerization interface causes dramatically reduced sedimentation coefficients, consistent with disruption of the quaternary structure. Source data

To further validate our experimental structure, we measured direct binding of cGAMP by these mutants using ITC. We found that mutations to the binding site reduced cGAMP binding by more than 5-fold and more than 25-fold for Y8A and F82A, respectively (Extended Data Fig. 8a,b). We also found that mutations to the hexamerization interfaces (L29, A66, A73 and A77) abrogated cGAMP binding (Extended Data Fig. 8a,b). Analytical ultracentrifugation confirmed that these mutations abolished oligomerization (Extended Data Fig. 8c). We also found that a relatively mild mutation (F33A), which is expected to reduce the buried surface area of the oligomer interface, destabilizes the complex, as indicated by the almost 50:50 mixture of hexameric and non-hexameric species observed with ultracentrifugation (Extended Data Fig. 8c). Consistent with this observation, we found that F33A binds cGAMP as well as wild-type Vs.4, but roughly half of the protein is inactive (incompetent fraction = 0.496). Additionally, the structure and mechanism of a Vs.4 homologue was independently confirmed in a contemporaneous study^18^. Together, these data provide strong evidence of our proposed structural model of the Vs.4–cGAMP complex.

## Discussion

**Extended Data Fig. 9:**
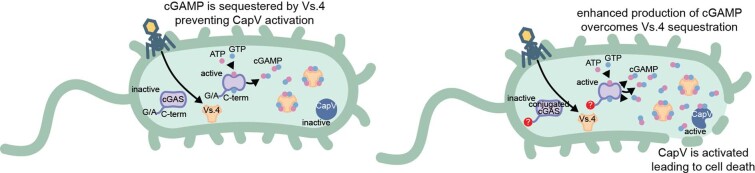
Model of the molecular antagonism between Vs.4 and cGAS conjugation. In response to phage infection, bacterial cGAS (purple) is activated and produces the second messenger cGAMP from ATP (red) and GTP (blue). cGAMP is bound by the phage encoded protein Vs.4 (orange), which acts as a cGAMP ‘sponge’ that sequesters cGAMP and prevents activation of the bacterial ‘suicide’ effector protein CapV. This allows the phage to propagate within the bacterial cell, as shown in the left panel. In contrast, when cGAS is conjugated to its biological target, enhanced cGAS activity produces abundant cGAMP that overpowers Vs.4 inhibition and activates the phospholipase activity of CapV. CapV activation disrupts the bacterial membrane and kills the bacterial cell before the phage replication cycle is complete, thereby preventing phage propagation.

Our data reveal an unusual ubiquitin-like conjugation system that plays an important role in the anti-phage defence of bacteria. Unlike ubiquitin and most other ubiquitin-like proteins, which are conjugated to other molecules through their C-terminal di-glycine, cGAS is conjugated to other molecules through a C-terminal glycine or alanine. This has been confirmed in an independent, concurrent study of Cap2 function^19^ The physiological targets of bacterial cGAS remain to be determined. Although our proteomics analyses showed that cGAS could be conjugated predominantly to the ε-amino group of a lysine residue on bacterial and phage proteins, it remains possible that the physiological target of cGAS is not a protein. For example, cGAS may be conjugated to a lipid in a manner similar to the lipidation of Atg8/LC3 in the autophagy system. Notably, the conservation of a C-terminal glycine or alanine of cGAS is found only in the bacterial CBASS operons that contain Cap2 and Cap3. It is likely that the cGAS conjugation system evolved during the arms race between bacteria and phages to defend against those phages that can antagonize cGAS signalling. The identification of Vs.4 in T4 phage provides an example of such an arms race. Vs.4 binds to cGAMP with a high affinity, thus sequestering cGAMP from its effector CapV, thereby enabling T4 phage to infect bacteria that do not produce enough cGAMP (Extended Data Fig. 9). As a counter measure, bacteria evolved the CBASS operons that contain Cap2 to catalyse ubiquitin-like conjugation of cGAS, thereby enhancing cGAMP production. These operons also contain Cap3, to render the conjugation reversible. However, the role of Cap3 is more complicated, as it is required for defence against some phages but not others. It is possible that Cap3 has additional targets besides deconjugation of cGAS. Future research should elucidate how this reversible ubiquitin-like conjugation of cGAS is regulated by phage infections, and in turn how cGAS conjugation impacts the arms race between bacteria and phages.

## Methods

### Cloning CBASS into *E. coli* MG1655

The regions containing CBASS operons together with approximately 1,000 base pairs (bp) of flanking sequence on either side were commercially synthesized based on the following sequences: *E. coli* TW11681 (GenBank: CP035855.1, 2375815–2381149), *V. cholerae* (GenBank: AE003852.1, 178939–84146), *P. aeruginosa*, (AXQR01000012.1, 1255165–1250237), *Enterobacter cloacae* (GenBank: JCKK01000002.1, 2262747–2268381). The constructs from *V. cholerae, P. aeruginosa* and *Enterobacter cloacae* were synthesized with Flag-tags at the N termini of their cGAS proteins. The products were assembled using Gibson assembly^20^ with a p15A origin and chloramphenicol resistance gene, which was amplified from the plysS plasmid with PCR. The empty vector control was assembled by connecting the ends of the plasmid backbone with PCR (to add homologous sequences) and Gibson assembly^20^. Point mutations and epitope insertions in these genes were created using site-directed mutagenesis^21^. Plasmids were transformed into DH5α cells (Lucigen, 60602) and the amplified plasmids were verified using Sanger sequencing. The plasmids were transformed into *E. coli* MG1655 (ATCC, 47076).

### Immunoblotting

Bacterial cultures were grown overnight in LB with the appropriate antibiotic. The cultures were diluted in LB media and grown to an optical density at 600 nm (OD_600_) of 1.0 ± 0.2. A volume equal to 1 OD_600_ of cells was pelleted by centrifugation and resuspended in 0.1 ml of chilled Dulbecco’s PBS (Sigma, D8537). The cell suspensions were sonicated at 4 °C. For some experiments DTT (1, 10 or 100 mM) or hydroxylamine (H_2_NOH; pH 7.0; 1, 10 or 100 mM) was added to the lysates and incubated at room temperature for 30 min, followed by addition of a sample buffer lacking reducing agent (Novex, LC2676). For immunoblotting of cGAS, the sample buffer was supplemented with 5% β-mercaptoethanol (BME). Samples were heated to 95 °C for 2 min, spun at 13,000 rpm on a table-top centrifuge and resolved by SDS–PAGE with Tris–glycine gels (4–12%, Novex). Separated proteins were transferred to a PVDF membrane (BioRad, 1704157). The membrane was blocked in TBST (20 mM Tris pH 7.5, 150 mM NaCl, 0.1% Tween-20) with 5% BSA before incubating with a 1:1,000 dilution anti-Flag antibody (Sigma) followed by 1:5,000 dilution anti-mouse IgG, HRP-linked secondary antibodies (Cell Signaling, 7076). Protein gel and immunoblot images were collected using BioRad ChemiDoc (Image Lab Touch). Data were analysed using BioRad Image Lab software.

### Phage cultivation

All phages used in this study were ordered from ATCC (catalogue nos): phages P1 (25404-B1), T2 (11303-B2), T4 (11303-B4), T5(11303-B5), T6 (11303-B6) and lambda (23724 B2). All phages were propagated in the *E. coli* MG1655 strain, which was also obtained from ATCC (47076). First, overnight cultures of the *E. coli* host cells were grown in LB (Miller) broth (Sigma, L3522) at 37 °C with shaking (220 rpm). Overnight cultures were diluted 100-fold in fresh LB media supplemented with 5 mM MgCl_2_, 5 mM CaCl_2_ and 0.1 mM MnCl_2_. The bacteria were inoculated with phage and cultured until the cells lysed completely. A few drops of chloroform were added to the media and the phage solution was centrifuged at 4,000 rpm for 10 min to remove the debris. The phages were transferred to a fresh tube, a few drops of chloroform were added and the phage stocks were stored at 4 °C. The titre of these stocks was determined using the small-drop plaque assay described below.

### Plaque assays

Small-drop plaque assays were used to determine phage stock titre and the efficiency of plating, as well as for library screening. To prepare the inoculated top agar, 120 µl of overnight bacterial culture was added to 5 ml of molten top agar (LB (Miller), 0.5% agar, 5 mM MgCl_2_, 5mM CaCl_2_ and 0.1 mM MnCl_2_ at 50 °C), quickly mixed and then poured onto a Petri dish. The plates were cooled at room temperature for approximately 30 min. To determine phage stock titre and the efficiency of plating, tenfold serial dilutions of phage stock were prepared in LB (Miller) broth and pipetted (5 µl per drop) with a multichannel pipette onto cooled top agar. The drops were dried for about 30 min before inverting the Petri dishes and incubating at 37 °C overnight. To quantify the plaque-forming units (PFU), individual plaques were counted from the lowest dilution drop with visible lysis. In some cases, individual plaques were not distinguishable, and rings of lysis were counted as 10 plaques. Sample sizes were chosen to reliably determine the differences between groups. Given the large effects, we performed experiments in triplicate or quadruplicate to demonstrate reproducibility. Blinding was not necessary for this study because the data had strong, reproducible effects and the raw data are reported in the manuscript. GraphPad Prism was used for data analysis.

### Expression of recombinant proteins

Bacterial cGAS (DNCV, residues 2–432) and Cap2 (residues 2–589) proteins from the CBASS operon from *E. coli* TW11681 were used for in vitro conjugation assays. Bacterial Cap3 (residues 1–156) and the tandem cGAS that was constructed from GAS (residues 2–432)–cGAS (residues 1–432) proteins from the CBASS operon from *E. coli* TW11681 were used for in vitro deconjugation assays. Vs.4 (residues 1–88) and Vs.4 point mutants from phage T4 were used for binding assays and crystallography. All proteins were expressed as Sumo fusions in BL21(DE3)pLysS *E. coli* transformed with a modified pET-SUMO vector containing the gene of interest. For all proteins, bacterial cells were grown in terrific broth (RPI) at 37 °C to an OD_600_ of 0.6 before inducing protein expression with 1 mM IPTG overnight at 18 °C. Bacterial cultures were collected by centrifugation and the pellets were frozen at −80 °C. Bacterial cGAS, Cap2 and Vs.4 were resuspended in binding buffer (20 mM Tris pH 7.4, 500 mM NaCl, 20 mM imidazole and 2 mM BME) with 0.2 mM phenylmethylsulfonyl fluoride (PMSF) as a protease inhibitor. Cell suspensions were lysed by sonication and centrifuged to remove cell debris. The clarified supernatant was applied to Ni-nitrilotriacetic acid agarose (Ni-NTA) resin that had been equilibrated in binding buffer and incubated at 4 °C with gentle rotation for about 1 h. The resin was washed three times with more than 10 ml of binding buffer. Bound protein was eluted with a 20 mM Tris pH 7.4, 300 mM NaCl, 200 mM imidazole. The Sumo–cGAS–cGAS protein was concentrated and frozen at −80 °C. The other proteins were diluted twofold with 20 mM Tris pH 7.4, 300 mM NaCl. DTT was added to a final concentration of 1 mM and SUMO tag was cleaved using ULP1 protease (0.01 mg protease per mg protein) overnight at 4 °C. The protein was buffer exchanged into 20 mM Tris pH 7.4 and 300 mM NaCl by serial spin filtration. To remove the SUMO tag and ULP1 (both of which have a His_6_ tag) from the expressed protein, the protein solution was passed through Ni-NTA resin equilibrated with 20 mM Tris pH 7.4 and 300 mM NaCl. For Cap2 and Cap3, the protein was concentrated and frozen at −80 °C. For bacterial cGAS and Vs.4, the protein was collected in the void column with size-exclusion chromatography on a S200 10/300 column with 20 mM Tris pH 7.4, 150 mM NaCl, 1 mM DTT. The purified proteins were concentrated and frozen at −80 °C. Purity was verified by SDS–PAGE and concentration was measured by UV absorbance at 280 nm.

To purify CapV (residues 2–356), the frozen bacterial pellet was thawed and resuspended in binding buffer (50 mM phosphate buffer pH 7.4, 300 mM NaCl, 20 mM imidazole, 10% glycerol and 2 mM BME) with 0.2 mM PMSF. Cell suspensions were lysed by sonication and centrifuged to remove cell debris. The clarified supernatant was applied to Ni-NTA resin that had been equilibrated in binding buffer and incubated at 4 °C with gentle rotation for about 1 h. The resin was washed three times with more than 10 ml of binding buffer. Bound protein was eluted with binding buffer supplemented with imidazole to a final concentration of 200 mM. The protein was diluted twofold with 50 mM phosphate buffer pH 7.4, 300 mM NaCl and 10% glycerol. DTT was added to a final concentration of 1 mM and SUMO tag was cleaved using ULP1 protease (0.01 mg protease per mg protein) overnight at 4 °C. The protein was buffer exchanged into 50 mM phosphate buffer pH 7.4, 300 mM NaCl and 10% glycerol by serial spin filtration. To remove the His_6_-SUMO tag and His_6_-ULP1 from CapV, the protein solution was passed through Ni-NTA resin equilibrated with 50 mM phosphate buffer pH 7.4, 300 mM NaCl and 10% glycerol. Purified CapV was concentrated and frozen at −80 °C. Purity was verified by SDS–PAGE and concentration was quantitated by UV absorbance at 280 nm.

### Measuring cGAMP in infected lysates

To measure cGAMP levels in T4 infected lysates, THP1 Lucia ISG cells (InvivoGen) were used. This cell line was authenticated using morphology and functional assay (expression of a secreted luciferase (Lucia) reporter gene under the control of an IRF-inducible promoter) and was not tested for mycoplasma contamination. To prepare the lysates, cultures of *E. coli* MG1655 cells containing empty vector or the *E. coli* CBASS operon with the indicated point mutation(s) were grown in LB with 5 mM MgCl_2_, 5 mM CaCl_2_ and 0.1 mM MnCl_2_ at 37 °C with shaking (220 rpm). When the cultures reached an OD_600_ of 1.0 they were infected with phage T4 at a multiplicity of infection of approximately 10 and cultured at 37 °C with shaking for 1 h (220 rpm). The samples were pelleted and the media was removed by decanting. The cell pellets were resuspended in 50 μl PBS, heated to 95 °C for 10 min and centrifuged at 20,000*g* for 10 min to remove the cell debris. From these lysates, 5 μl was added to 2.25 × 10^5^ THP1 Lucia ISG reporter cells in 45 μl of RPMI 1640 medium with 10% FBS supplemented with 50 ng ml^−1^ recombinant perfringolysin O (PFO). The samples were then incubated for 16 h at 37 °C. Twelve microlitres of this culture were transferred into 45 μl of a buffer containing 1 μM coelenterazine (Gold Biotechnology), 50 mM Tris–HCl, pH 7.0, 50 mM NaCl, 20 mM EDTA and 20% glycerol. Luminescence was measured in an opaque 96-well plate with a CLARIOstar microplate reader and CLARIOstar plate reader software (BMG LABTECH). Pure cGAMP (0.01 to 30 μM) was used to obtain a standard curve.

The previously described fluorogenic assay for CapV^8^ was used to quantitate 3′,3′-cGAMP in P1 infected lysates. To prepare the lysates, cultures of *E. coli* MG1655 cells containing empty vector, the *E. coli* CBASS operon and the *E. coli* CBASS operon containing a point mutation (cGAS ΔG432) were grown in LB with 5 mM MgCl_2_, 5 mM CaCl_2_ and 0.1 mM MnCl_2_ at 37 °C with shaking (220 rpm). When the cultures reached an OD_600_ of 1.0 they were infected with phage P1 at a multiplicity of infection of 2 and cultured at 37 °C with shaking (220 rpm). At the time of infection 100 μl was taken from each culture as the 0 min time point and frozen in liquid nitrogen. Additional samples were taken from each culture at 10 min intervals starting at 40 min and ending at 100 min post infection, and frozen in liquid nitrogen. The samples were heated to 95 °C for 10 min, sonicated and centrifuged at 20,000*g* for 10 min to remove the cell debris. From these lysates, 10 µl was mixed with 10 µl of purified recombinant CapV to measure CapV phospholipase activity as described below.

### CapV inhibition assay

cGAMP (1 µM) was mixed with 10 µM of the indicated recombinant Vs.4 protein in a total volume of 10 µl, which was then mixed with 10 µl of purified recombinant CapV protein (2 µM in PBS and 1% NP-40) and incubated at room temperature for 10 min. The fluorogenic substrate resorufin butyrate (Cayman Chemical, 19592) was dissolved in DMSO and diluted to 64 µM in PBS and 1% NP-40 before addition (30 µl) to each sample. Plates were developed at room temperature in Grenier 96-well plates (655083) until a strong fluorescence signal developed (approximately 30 min), which was read with a CLARIOstar plate reader (BMG LABTECH) with excitation and absorption wavelengths of 560 nm and 595 nm, respectively.

### Bacteriophage mutagenesis and screening

Construction of the T4 phage library followed a previously established protocol^22^. In brief, a stock of approximately 10^11^ PFU of T4 phage was cultivated (see ‘Phage cultivation’) and buffer exchanged to phage buffer (50 mM Tris pH 7.5, 100 mM MgCl_2_, 10 mM NaCl) by spin filtration using an Amicon filter (100 kDa MCO; ACS510024) and concentrated into 1 ml. The phages were chemically mutagenized by mixing 0.2 ml of concentrated phage stock with 0.4 ml of the phosphate–EDTA buffer (500 mM KH_2_PO_4_ pH 6, 5 mM EDTA), 0.8 ml sterile double-distilled water, 0.2 ml of MgSO_4_ (100 mM), 0.4 ml 1 M hydroxylamine solution (0.175 g HONH_2_·HCl, 0.28 ml 4 M NaOH and distilled water to 2.5 ml). A control reaction to monitor the effect of the mutagen on viability was done by replacing hydroxylamine solution with PBS. The reactions were incubated at 37 °C for about 48 h. The viability of the mutagenized and control phage stocks was determined using the small-drop plaque assay (described above) and the mutagenized phage was found to have a viability of 0.1% that of the control, indicating sufficient mutagenesis. The mutagenized phage was dialysed for 24 h at 4 °C with phage buffer in a Pierce Slide-A-Lyzer cassette with a buffer change mid-way through.

To isolate individual mutants, a tenfold dilution series of phage was made in 120 μl aliquots of *E. coli* MG1655, which was cultured overnight. The phage/bacteria solutions were mixed with molten top agar (LB (Miller), 0.5% agar, 5 mM MgCl_2_, 5 mM CaCl_2_ and 0.1 mM MnCl_2_ at 50 °C), quickly mixed and then poured onto a Petri dish. The plates were cooled at room temperature for about 30 min before incubating at 37 °C overnight. Isolated plaques were picked using sterile toothpicks and suspended in LB in 96-well plates. These phage-containing solutions were pipetted onto the surface of top agar (see above) that had been inoculated with *E. coli* MG1655 harbouring no CBASS (empty vector), wild-type CBASS (CBASS) or CBASS with inactivating Cap2 mutations (CBASS Cap2 C493A/C496A). Mutants that formed plaques only on the empty vector plates were picked by toothpick and repeated to confirm the phenotype. Clones with reproducible phenotypes were cultivated and titrated to determine their efficiency of plating on all three strains of bacteria used in this screen (see above).

To determine the genotypes of the mutants of interest, phage DNA was isolated using a kit (Norgen Biotek, NC0690454) according to the manufacturer’s instructions, including the optional DNase treatment step. The entire genomes of these phage mutants and the parental wild-type T4 phage were determined using Illumina Novaseq 150 bp paired-end reads. Sequencing data are available in the Sequence Read Archive (SRA) under BioProject PRJNA931786. Reads were filtered by quality control and assembled using SeqMan NGen (DNASTAR).

To validate the top hit from this screen, the *vs.4* gene from phage T4 was knocked out using pCas9 (Addgene, 42876) and pCRISPR (Addgene, 42875)^23,24^. CHOPCHOP^25^ was used to design crRNA targeting *vs.4*. These sequences were cloned into pCRISPR along with a cassette for genetic insertion into the T4 genome at the *vs.4* locus. This cassette included approximately 500 base pairs of homology to *vs.4* on both sides of a reporter gene (*lacZ* cloned from pUC19). An overnight culture (100 µl) of *E. coli* DH5α containing pCRISPR (genomic RNA + donor) was inoculated with approximately 10^5^ wild-type T4 phage and added to molten 0.5% LB top agar containing 5 mM MgCl_2_, 5 mM CaCl_2_ and 0.1 mM MnCl_2_ with the appropriate antibiotics, and then mixed and poured onto an LB plate for overnight growth at 37 °C. The following day, plaques were picked and re-plated on *E. coli* MG1655 to remove background from the plasmids. Plaques from this plate were screened by PCR followed by separation of DNA on an agarose gel. Vs.4 knockout was confirmed using Sanger sequencing.

### X-ray crystallography

Crystals of Vs.4 in complex with 3′3′-cGAMP (Invivogen) were grown in hanging drops over a reservoir containing 7% PEG8000, 0.1 M imidazole pH 6.3 and 0.2 mM calcium acetate at room temperature. cGAMP was spiked into the protein solution at a 1.2:1 molar ratio. The hanging drops contained 1 μl of complex mixed with 1 μl of reservoir solution. Crystals were cryoprotected by transferring into reservoir solution supplemented with 17.5% ethylene glycol before flash freezing. Diffraction data were collected at the Advanced Photon Source at the Argonne National Laboratory, beamline 19-ID with a wavelength of 1.0448 Å at 100 K. The data were integrated and scaled to 2.0 Å using HKL-3000 and phased by molecular replacement with PHASER using a predicted structure of Vs.4 that was created using AlphaFold^26–29^ (Extended Data Fig. 7a). The structure was refined with iterative rounds of refinement and model building using PHENIX and Coot^29,30^. The Ramachandran statistics for the final model were 99.01% favoured, 0.99% allowed and 0.00% outliers. The clashscore for the final model was 6 and the model had 1.4% side chain outliers. PISA^31^ analysis (accessed online on the PDBePISA website) was used to confirm the quaternary structure assignments. PyMOL was used to create figures.

### In vitro thioester assay

To reconstitute the covalent attachment of cGAS to Cap2 in vitro, these recombinant proteins (purification described above) were mixed in 50 mM HEPES pH 7.5, 300 mM NaCl, 5 mM TCEP (pH adjusted to 7.0), 1 mM CaCl_2_, 20 mM MgCl_2_ and 10 mM ATP (pH adjusted to 7.0). The mixture was incubated at 37 °C for 30 min. The reaction was quenched by adding LDS sample buffer (NuPAGE) and heating at 70 °C for 10 min. Proteins in the solutions were separated by SDS–PAGE in the absence of reducing agent and stained with Coomassie blue. To test the activity of the conjugates formed in vitro, cGAS, Cap2 and Cap3 were incubated in 50 mM HEPES pH 7.5, 300 mM NaCl, 5 mM TCEP (pH adjusted to 7.0), 1 mM CaCl_2_, 20 mM MgCl_2_ and 10 mM ATP (pH adjusted to 7.0) and incubated at 37 °C for 30 min. The samples were desalted with Zeba Spin Desalting Columns, 7K MWCO, 0.5 ml (Thermo Scientific) and buffer exchanged into PBS with 10 mM MgCl_2_ according to the manufacturer’s instructions. The samples were diluted in a threefold dilution series before ATP and GTP were added to a final concentration of 1 mM of each. The reaction was incubated 37 °C for 30 min before the reactions were quenched by heating at 95 °C for 2 min. cGAMP was measured using the THP1 Lucia ISG cells as described above in ‘Measuring cGAMP in infected lysates’.

### In vitro Cap3 assay

To test if Cap3 functions as a cGAS isopeptidase in vitro, recombinant Sumo–cGAS–cGAS or FLAG IP eluates were mixed in 20 mM HEPES pH 7.4, 100 mM NaCl, 20 mM MgCl_2_, 20 mM ZnCl_2_, 1 mM DTT, with and without recombinant Cap3. The mixture was incubated at 37 °C for 120 min. For western blot analysis, the reaction was quenched by adding LDS sample buffer (NuPAGE) and heating at 80 °C for 10 min. Proteins in the solutions were separated by SDS–PAGE and stained with Coomassie blue or immunoblotted with an anti-FLAG antibody. For cGAS activity measurements, FLAG IP eluates treated with Cap3 or a buffer-only control were diluted in a threefold dilution series before ATP and GTP were added to a final concentration of 1 mM of each. The reaction was incubated 37 °C for 120 min before the reactions were quenched by heating at 95 °C for 2 min. cGAMP was measured using the THP1 Lucia ISG cells as described above in ‘Measuring cGAMP in infected lysates’.

### Protein alignments

WebLogo^32^ was used to display the sequence logo of the C-terminal sequences of cGAS from previously identified CBASS operons^12^.

### Phylogenetic analysis

Homologues of Vs.4 were identified using HHpred^33^ searching the PHROGs_v4 database^34^. PHROG 717 was found to contain Vs.4 homologues. Sequences from this PHROG were aligned using Clustal Omega^35^ and a phylogenetic tree was generated using the default settings. iTOL: Interactive Tree of Life^36^ was used to display the phylogenetic tree. WebLogo^32^ was used to display a multiple sequence alignment.

### Isothermal calorimetry

The binding affinity between Vs.4/Vs.4 mutants and 3′3′-cGAMP was determined with a VP-ITC microcalorimeter (GE Healthcare) using MicroCal ITC200 control software (GE). Vs.4 was dialysed against 3 l of 20 mM Tris, 150 mM NaCl overnight. cGAMP (Invivogen) was resuspended in the buffer used for dialysis. The titrations were performed at 20 °C. A total of 21 injections were performed with 2 min spacing time. The titration traces were integrated by NITPIC, and then the curves were fitted using the concentration of cGAMP binding sites with SEDPHAT^37,38^. The figures were prepared using GUSSI^39^.

### Analytical ultracentrifugation

The oligomerization state of Vs.4 and 3′3′-cGAMP was determined with an Optima XL-I analytical ultracentrifuge (Beckman–Coulter) at 20 °C using the Rayleigh interferometer and the software ProteomeLab (Beckman–Coulter). Vs.4 was dialysed against 3 l of 20 mM Tris, 150 mM NaCl overnight. cGAMP (Invivogen) was resuspended in the buffer used for dialysis. The reference sector was filled with 90 µM cGAMP in 20 mM Tris, 150 mM NaCl. The sample sectors were filled with 45 µM, 15 µM or 4.5 µM Vs.4 in 90 µM cGAMP in 20 mM Tris, 150 mM NaCl. The samples were centrifuged at 50,000 rpm, with scans collected until solute migration could be no longer be observed. The curves were fitted using SEDFIT^40^. The figures were prepared using GUSSI^39^. The oligomerization states of the Vs.4 mutants were determined by preparing the mutants as described above. For these experiments, the sectors were filled with 15 µM of each mutant protein in 20 mM Tris and 150 mM NaCl.

### Protein identification by mass spectrometry

To identify the protein covalently modified to Cap2, samples of whole-cell lysates from bacterial cultures with wild-type and inactive Cap2 (C493A/C496A) were obtained by growing these cultures overnight in LB with the appropriate antibiotic. The cultures were diluted in LB media and grown to an OD_600_ of 1.0. A volume equal to 1 OD_600_ of cells was pelleted by centrifugation and resuspended in 0.1 ml of chilled Dulbecco’s PBS (Sigma, D8537). The cell suspensions were sonicated at 4 °C. Sample buffer (without reducing agent) (Novex, LC2676) was added to each sample and the samples were boiled.

To identify cGAS conjugation sites, cells were grown in LB (Miller) supplemented with 5 mM MgCl_2_, 5 mM CaCl_2_ and 0.1 mM MnCl_2_ at 37 °C to an OD_600_ of 1 and were collected immediately by centrifugation at 4,000*g* for 10 min at 4 °C, or were infected by T4 phage at multiplicity of infection of about 10 and incubated at 37 °C for 40 min before collecting by centrifugation. The cell pellets were resuspended in an equal mass of lysis buffer (50 mM Tris pH 7.4, 250 mM NaCl, 0.5% Chaps), and were frozen by dripping into liquid nitrogen, before being cryogenically disrupted using the Retsch MM301 mixer mill by 10 steps of 2.5 min at 30 Hz with intermittent cooling in liquid nitrogen. The frozen cell powders were suspended in lysis buffers, homogenized for 10 s with a PT 10–35 Polytron (Kinematica) and centrifuged for 10 min at 10,000*g* at 4 °C. The supernatant was used for affinity purification with anti-DYKDDDDK magnetic agarose (Pierce, A36797). Protein samples were eluted by boiling in LDS sample buffer (NuPAGE).

Protein samples were separated on 10% NuPAGE Tris–glycine gels (Thermo Scientific) and visualized with SimplyBlue SafeStain (Thermo Scientific). Protein bands were excised and de-stained in 50 mM ammonium bicarbonate (ABC) and 50% acetonitrile (ACN) for 10 min. Gel slices were dehydrated in 100% ACN and rehydrated in 50 mM ABC plus 5 mM DTT for 30 min. Iodoacetamide (50 mM) was added for 60 min. Gel pieces were dehydrated, rehydrated in ABC and additionally dehydrated before overnight incubation with 12.5 ng μl^−1^ trypsin (Promega) in 50 mM ABC at 37 °C. The resulting peptides were extracted in 1% formic acid (FA) at 25 °C for 4 h and then in 0.5% FA/0.5% ACN for 2 h. After acidification in 1% formic acid, peptide mixtures were further purified with C18 Zip-tip (Millipore) and analysed by nano liquid chromatography–mass spectrometry (nLC–MS).

Peptide mixtures were separated on an in-house packed C18 column in silica capillary emitters (resin: 100 Å, 3 µm, MICHROM Bioresources; column: 100 µm internal diameter, 100 mm resin length). A Dionex Ultimate 3000 nanoLC system (Thermo Scientific) provided the LC gradient with 0.1% formic acid as mobile phase A and 0.1% formic acid in acetonitrile as mobile phase B. The following gradient was used: 2% B at 0–15 min, 30% B at 81 min, 35% B at 85 min, 40% B at 87 min, 60% B at 95 min, 80% B at 96–107 min and 2% B at 108–120 min. Flow rate was 600 nl min^−1^ at 0–13.5 min and 250 nl min^−1^ at 13.5–120 min.

Peptide eluents were sprayed online with a nano-electrospray ion source (Thermo Scientific with Xcalibur software) at a spray voltage of 1.5 kV and a capillary temperature of 250 °C. High-resolution MS analysis was performed on a QExactive HF-X quadrupole-orbitrap hybrid mass spectrometer (Thermo Scientific), operating in data-dependent mode with dynamic exclusion of 30 s. Full-scan MS was acquired at an *m*/*z* range of 300–1,650, resolution of 60,000 and automatic gain control target of 3 × 10^6^ ions. The top 15 most intense ions were subsequently selected for higher energy collisional dissociation fragmentation at a resolution of 15,000, collision energy of 30 eV and automatic gain control target of 1 × 10^5^.

Peptide-spectrum matches were performed by the SEQUEST algorithm in Proteome Discoverer (Thermo Scientific), using the *E. coli* strain K12 proteome database (Uniprot, UP000000625), the T4 phage proteome database (Uniprot, UP000009087), *E.coli* CBASS proteins (Cap2, P0DTF2 ; cGAS, P0DTF0; CapV, P0DTE9; Cap3, P0DTF3) and common contaminants. Static modification: carbamidomethylation on cysteines; variable modifications: methionine oxidation, glutamine or asparagine deamination and the cGAS C-terminal remnant after trypsin digestion (Thr-Met-Val-Ser-Gly with a delta mass of 476.21735 Da); precursor mass error: 10 ppm; fragment mass error: 0.05 Da; maximum miscleavage: 2; peptide false discovery rate: 1%.

### Reporting summary

Further information on research design is available in the Nature Portfolio Reporting Summary linked to this article.

## Online content

Any methods, additional references, Nature Portfolio reporting summaries, source data, extended data, supplementary information, acknowledgements, peer review information; details of author contributions and competing interests; and statements of data and code availability are available at 10.1038/s41586-023-05862-7.

### Supplementary information

Supplementary Information Supplementary Fig. 1 and Tables 1 and 2. Supplementary Fig. 1: Uncropped and unedited versions of all gels used in this study. Supplementary Table 1: Top 20 cGAS-conjugated proteins. A complete list of protein modifications identified by IP-MS is available in Source Data Extended Data Fig. 4. Supplementary Table 2: The 20 most-enriched cGAS-conjugated proteins in cells infected with phage T4. A complete list of protein modifications identified by IP-MS is available in Source Data Extended Data Fig. 4.

Reporting Summary

### Source data

Source Data Fig. 1

Source Data Fig. 2

Source Data Fig. 3

Source Data Extended Data Fig. 1

Source Data Extended Data Fig. 2

Source Data Extended Data Fig. 3

Source Data Extended Data Fig. 4

Source Data Extended Data Fig. 6

Source Data Extended Data Fig. 8

## Figures and Tables

**Extended Data Table 1:**
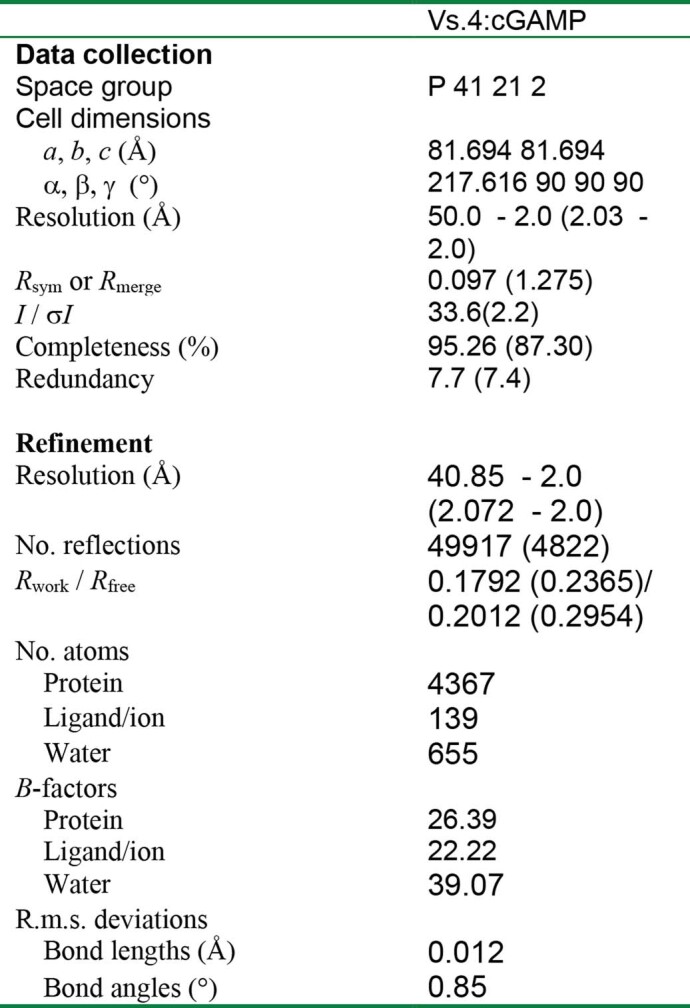
Data collection and refinement statistics

*Structure was determined from the data of a single crystal.

## Data Availability

The structure of Vs.4 from T4 phage in complex with cGAMP is publicly available at the RCSB Protein Data Bank (PDB 7UQ2). The *E. coli* strain K12 proteome database that was used in this study can be accessed at Uniprot (UP000000625). The T4 phage proteome database that was used in this study can be accessed at Uniprot (UP000009087). Vs.4 homologues can be accessed at the PHROGs database (PHROG 717). Sequence Read Archive (SRA) under BioProject PRJNA931786. GenBank accessions for the CBASS operons used in this study are found in the Methods. Materials, including strains and plasmids, are available upon reasonable request. Source data are provided with this paper.
