# Supplementary material for: Ubiquitin-like conjugation by bacterial cGAS enhances anti-phage defence
Source: Nature. 2023 Feb 27;616(7956):326–31. doi: 10.1038/s41586-023-05862-7 (PMC10097602; doi:10.1038/s41586-023-05862-7)
Supplement: Supplementary file 1 — Supplementary Fig. 1 and Tables 1 and 2. Supplementary Fig. 1: Uncropped and unedited versions of all gels used in this study. Supplementary Table 1: Top 20 cGAS-conjugated proteins. A complete list of protein modifications identified by IP-MS is available in Source Data Extended Data Fig. 4. Supplementary Table 2: The 20 most-enriched cGAS-conjugated proteins in cells infected with phage T4. A complete list of protein modifications identified by IP-MS is available in Source Data Extended Data Fig. 4. [file 41586_2023_5862_MOESM1_ESM.pdf]

nature portfolio

https://doi.org/10.1038/s41586-023-05862-7

Supplementary information

Ubiquitin-like conjugation by bacterial cGAS enhances anti-phage defence

In the format provided by the authors and unedited

Used in Fig. 1b

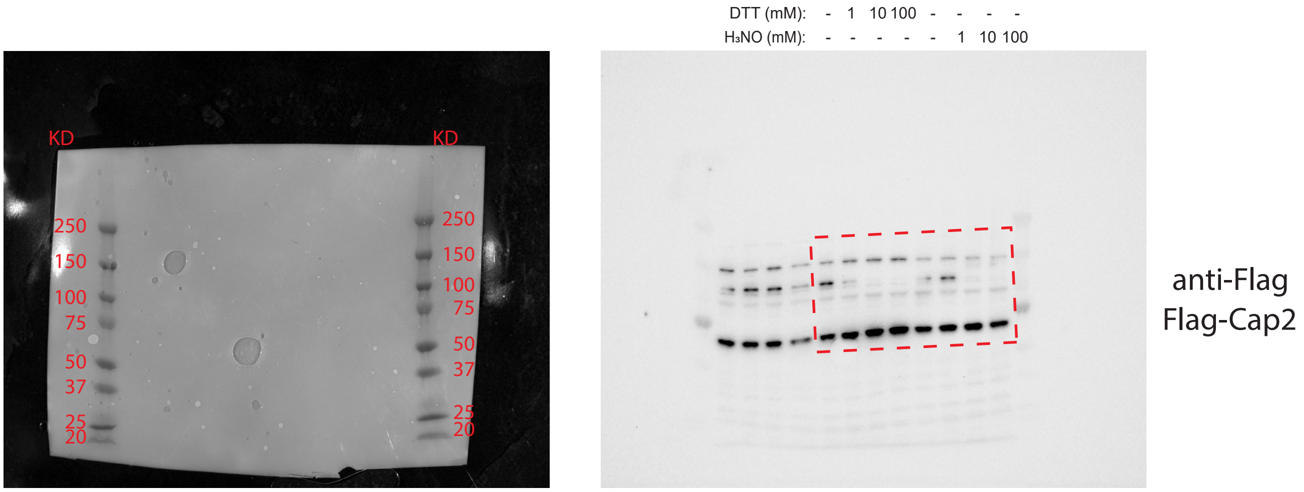

Used in Fig. 1e

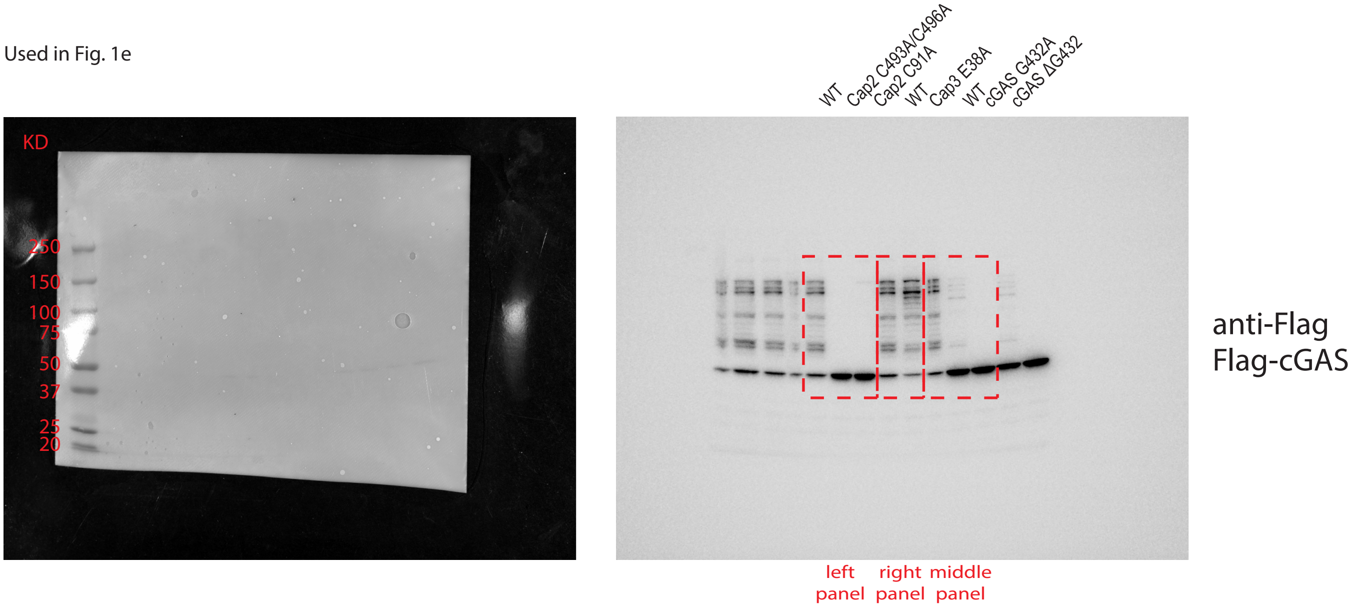

Used in Fig. 1f

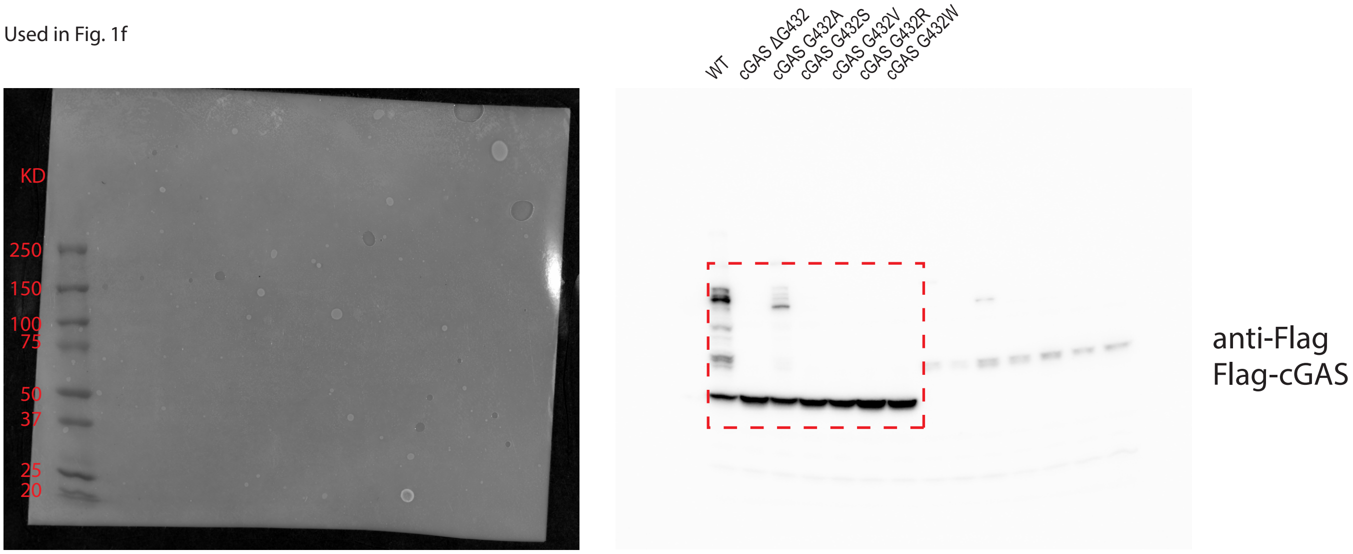

Used in Fig. 1g

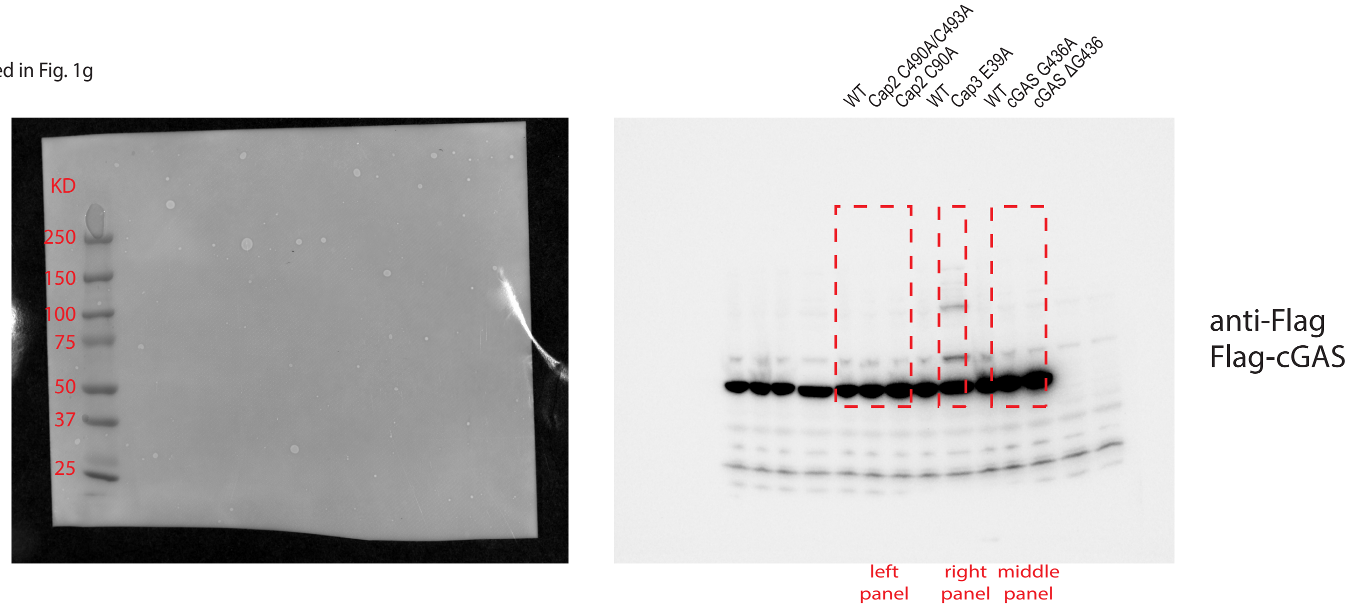

Used in Fig. 1h

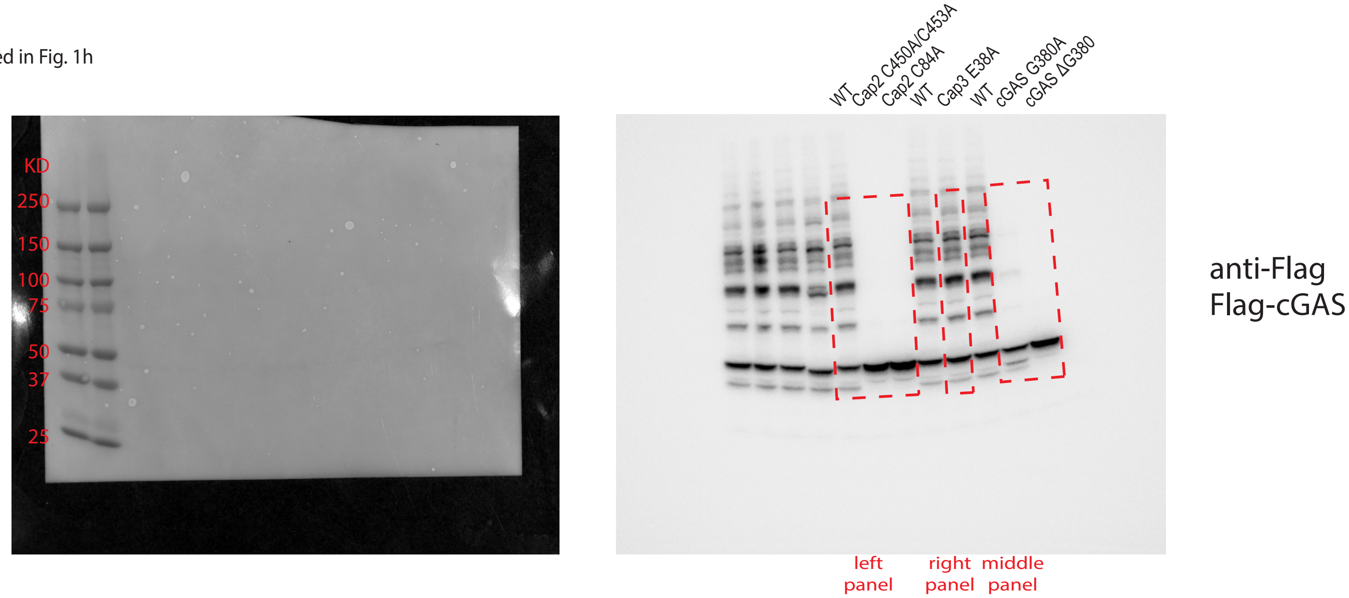

Used in Fig. 1i

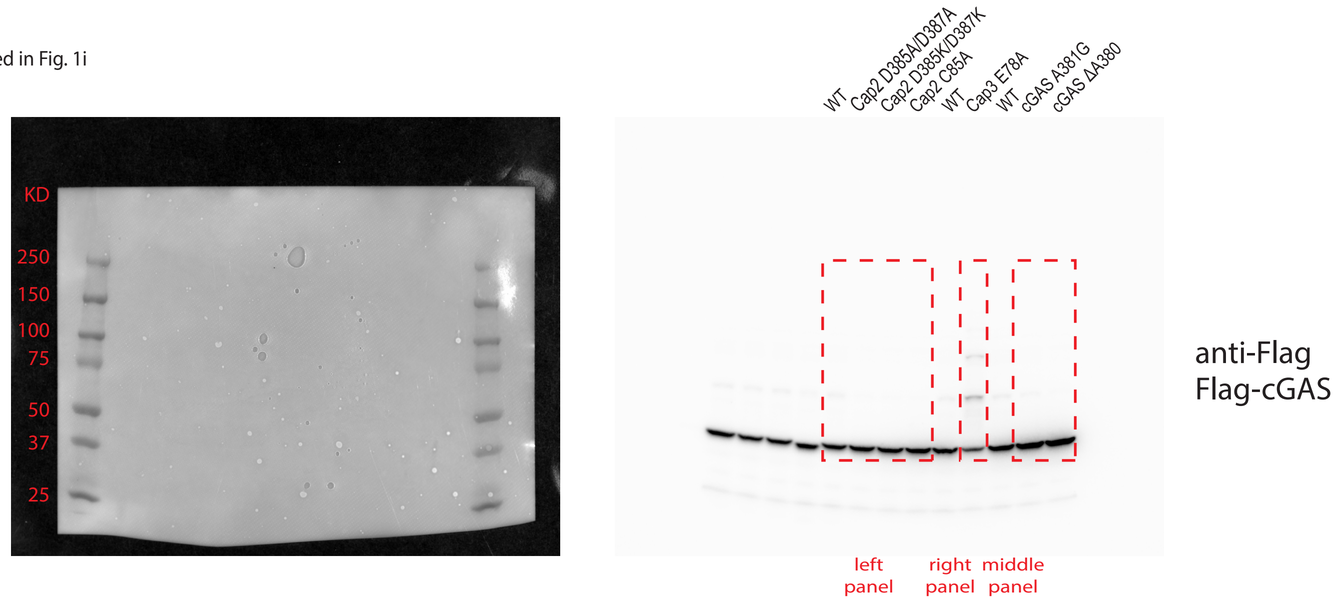

Used in Fig. 1j

cGAS: + - +
Cap2: - + +

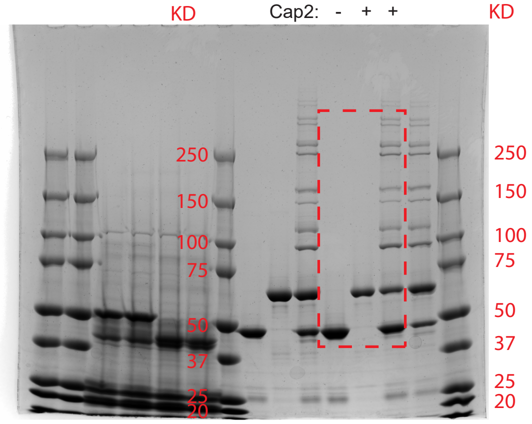

Used in Extended Data Fig. 1a

Sumo-cGAS-cGAS: - + +
Cap3: + - +

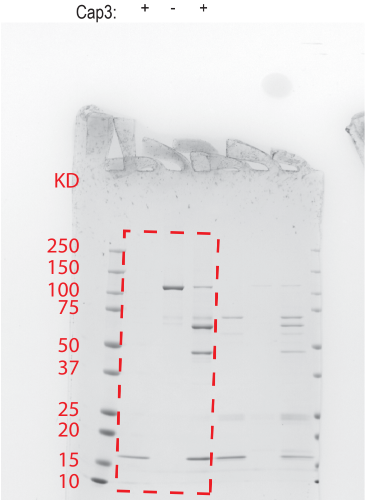

Used in Extended Data Fig. 1d

flag-IP: + +
Cap3: - +

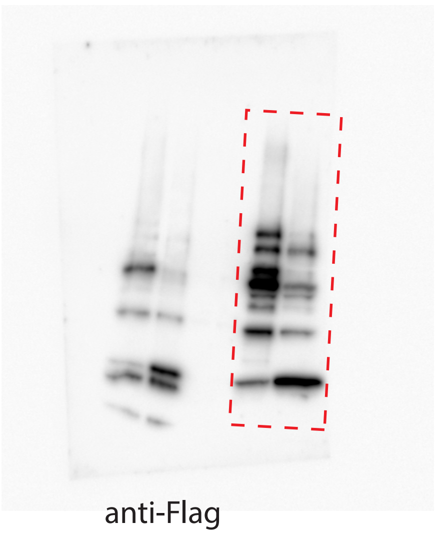

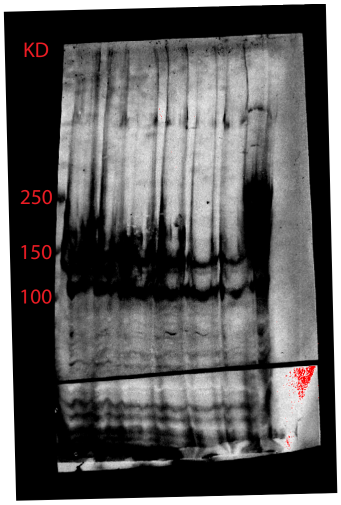

anti-Flag
Flag-cGAS

other half of membrane
with visible mw markers

Used in Extended Data Fig. 1f

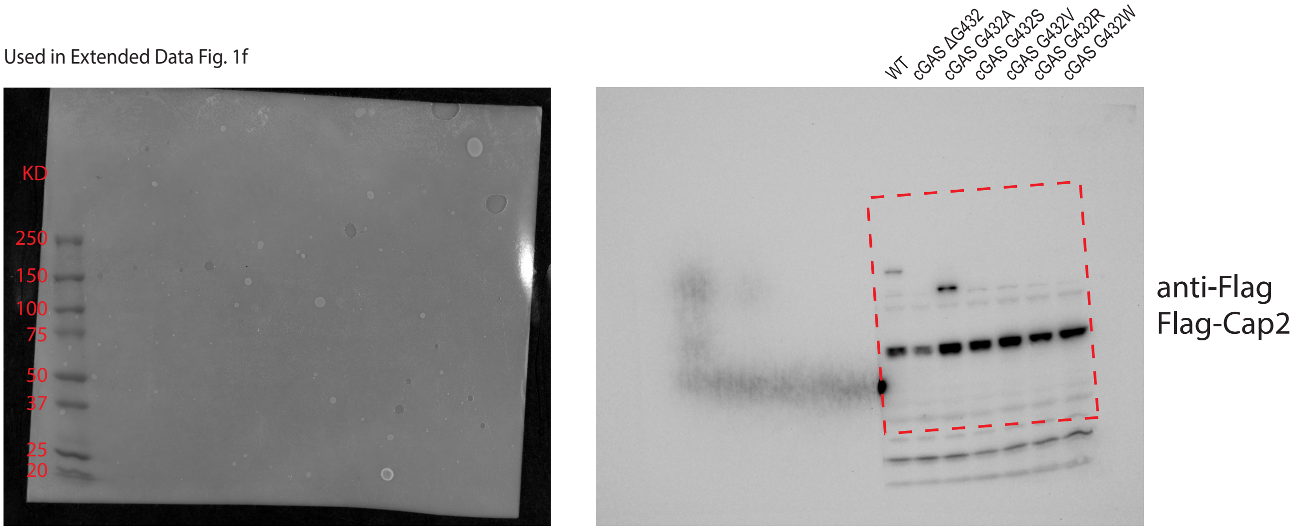

Used in Extended Data Fig. 1g

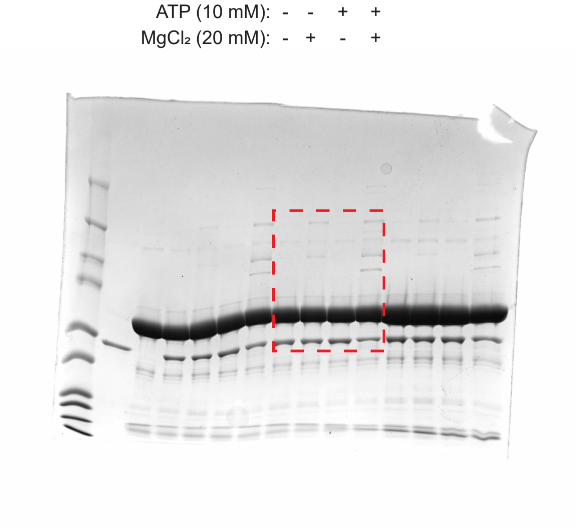

Used in Extended Data Fig. 3a

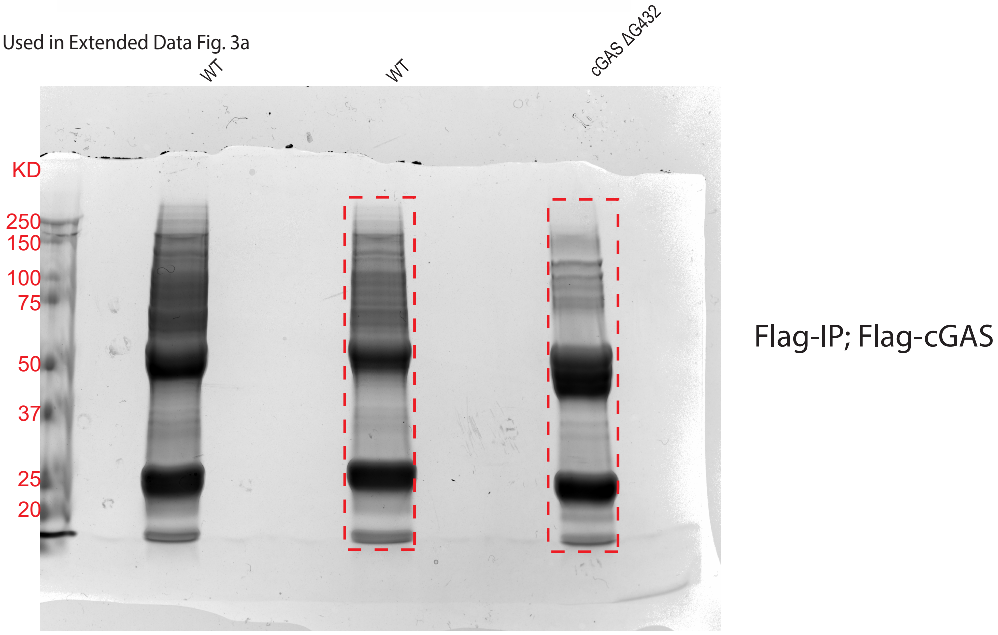

Supplementary Fig. 1 uncropped and unedited versions of all gels used in this study.

Supplementary Table 1| Top 20 cGAS conjugated proteins.

Accession	Description	Gene	Coverage [%]	# Peptides	MW [kDa]	Score Sequest HT	Abundance		TMVSG sites
							ΔG432	WT	
P06959	Dihydrolipoyllysine-residue acetyltransferase	aceF	91	74	66	6613	181	86	1
P09373	Formate acetyltransferase 1	pflB	93	86	85	5254	10	114	2
**E1E2**	**ThiF family adenylyltransferase**	**E1E2**	90	75	67	3889	6	135	5
P0A6F5	60 kDa chaperonin	groL	92	50	57	2781	2	169	2
P07395	Phenylalanine--tRNA ligase beta subunit	pheT	85	73	87	2696	3	152	1
P36683	Aconitate hydratase 2	acnB	89	75	93	2528	5	141	1
P0A8M3	Threonine--tRNA ligase	thrS	85	64	74	1719	6	131	2
P09152	Respiratory nitrate reductase 1 alpha chain	narG	83	88	140	1642	67	101	1
P0A853	Tryptophanase	tnaA	89	42	53	1596	28	119	4
P0A6M8	Elongation factor G	fusA	81	49	78	1444	52	106	2
P0CE48	Elongation factor Tu 2	tufB	86	29	43	1149	6	110	1
P0A9B2	Glyceraldehyde-3-phosphate dehydrogenase A	gapA	92	39	36	1037	29	125	4
P05055	Polyribonucleotide nucleotidyltransferase	pnp	69	48	77	960	124	94	1
P08839	Phosphoenolpyruvate-protein phosphotransferase	ptsI	86	48	64	752	15	94	1
P0A799	Phosphoglycerate kinase	pgk	94	37	41	613	4	126	2
P0A9C5	Glutamine synthetase	glnA	85	34	52	612	2	148	1
P00961	Glycine--tRNA ligase beta subunit	glyS	85	58	77	605	51	113	1
P0A6P9	Enolase	eno	86	36	46	597	1	144	1
P0A6T1	Glucose-6-phosphate isomerase	pgi	80	44	62	576	1	128	2
P00957	Alanine--tRNA ligase	alaS	74	57	96	546.36	31	138	1

Supplementary Table 2| 20 most enriched cGAS conjugated proteins in cells infected with phage T4.

Description	Sum PEP Score	Coverage [%]	Abundance Ratio: (T4 infected) / (Uninfected)	Found in Sample: Uninfected	Found in Sample: T4 infected	Modifications
Uncharacterized 9.5 kDa protein in dexA-dda intergenic region OS=Enterobacteria phage T4 OX=10665 GN=y00H PE=4 SV=1	22.617	70	100	Not Found	High	TMVSG [K5]
Fibritin OS=Enterobacteria phage T4 OX=10665 GN=wac PE=1 SV=5	111.34	69	21.028	High	High	TMVSG [K100]
50S ribosomal protein L4 OS=Escherichia coli (strain K12) GN=rplD PE=1 SV=1	64.668	59	18.773	High	High	TMVSG [K6]
50S ribosomal protein L9 OS=Escherichia coli (strain K12) GN=rplI PE=1 SV=1	48.507	78	12.396	High	High	TMVSG [K22]
Protein-export protein SecB OS=Escherichia coli (strain K12) GN=secB PE=1 SV=1	27.533	54	4.542	High	High	TMVSG [K19]
Glycerol kinase OS=Escherichia coli (strain K12) GN=glpK PE=1 SV=2	220.956	72	2.741	High	High	TMVSG [K143]
Citrate synthase OS=Escherichia coli (strain K12) GN=gltA PE=1 SV=1	181.654	85	2.078	High	High	TMVSG [K38]
60 kDa chaperonin OS=Escherichia coli (strain K12) GN=groL PE=1 SV=2	527.371	90	1.983	High	High	TMVSG [K7]
Lipid A export ATP-binding/permease protein MsbA OS=Escherichia coli (strain K12) GN=msbA PE=1 SV=1	5.504	5	1.943	Peak Found	High	TMVSG [K528]
3-oxoacyl-[acyl-carrier-protein] synthase 2 OS=Escherichia coli (strain K12) GN=fabF PE=1 SV=2	62.461	65	1.832	High	High	TMVSG [K49]
Ribosomal protein S12 methylthiotransferase RimO OS=Escherichia coli (strain K12) GN=rimO PE=1 SV=1	46.233	37	1.752	High	High	TMVSG [K19]
Alkyl hydroperoxide reductase subunit C OS=Escherichia coli (strain K12) GN=ahpC PE=1 SV=2	171.942	90	1.603	High	High	TMVSG [K17]
Glycine cleavage system H protein OS=Escherichia coli (strain K12) GN=gcvH PE=1 SV=2	10.867	27	1.542	High	High	TMVSG [K12]
Galactitol-specific phosphotransferase enzyme IIA component OS=Escherichia coli (strain K12) GN=gatA PE=4 SV=1	48.243	90	1.41	High	High	TMVSG [K29]
Acetyl-coenzyme A carboxylase carboxyl transferase subunit alpha OS=Escherichia coli (strain K12) GN=accA PE=1 SV=2	57.406	56	1.303	High	High	TMVSG [K53]
Elongation factor Tu 1 OS=Escherichia coli (strain K12) GN=tufA PE=1 SV=1	216.33	90	1.294	High	High	TMVSG [K38; K177]
Elongation factor Tu 2 OS=Escherichia coli (strain K12) GN=tufB PE=1 SV=1	216.33	90	1.294	High	High	TMVSG [K38; K177]
Lactaldehyde dehydrogenase OS=Escherichia coli (strain K12) GN=aldA PE=1 SV=2	138.001	76	1.288	High	High	TMVSG [K356]
Lysine--tRNA ligase OS=Escherichia coli (strain K12) GN=lysS PE=1 SV=2	219.952	77	1.116	High	High	TMVSG [K82]
Bifunctional purine biosynthesis protein PurH OS=Escherichia coli (strain K12) GN=purH PE=1 SV=1	72.007	45	1.069	High	High	TMVSG [K17]
