# Supplementary material for: Ubiquitin-like conjugation by bacterial cGAS enhances anti-phage defence
Source: Nature. 2023 Feb 27;616(7956):326–31. doi: 10.1038/s41586-023-05862-7 (PMC10097602; doi:10.1038/s41586-023-05862-7)
Supplement: Supplementary file 2 — Reporting Summary [file 41586_2023_5862_MOESM2_ESM.pdf]

nature portfolio

Corresponding author(s): Zhijian "James" Chen

Last updated by author(s): Feb 6, 2023

Reporting Summary

Nature Portfolio wishes to improve the reproducibility of the work that we publish. This form provides structure for consistency and transparency in reporting. For further information on Nature Portfolio policies, see our Editorial Policies and the Editorial Policy Checklist.

Statistics

For all statistical analyses, confirm that the following items are present in the figure legend, table legend, main text, or Methods section.

n/a	Confirmed	
☐	☒	The exact sample size (*n*) for each experimental group/condition, given as a discrete number and unit of measurement
☐	☒	A statement on whether measurements were taken from distinct samples or whether the same sample was measured repeatedly
☐	☒	The statistical test(s) used AND whether they are one- or two-sided<br>*Only common tests should be described solely by name; describe more complex techniques in the Methods section.*
☒	☐	A description of all covariates tested
☒	☐	A description of any assumptions or corrections, such as tests of normality and adjustment for multiple comparisons
☐	☒	A full description of the statistical parameters including central tendency (e.g. means) or other basic estimates (e.g. regression coefficient) AND variation (e.g. standard deviation) or associated estimates of uncertainty (e.g. confidence intervals)
☐	☒	For null hypothesis testing, the test statistic (e.g. *F*, *t*, *r*) with confidence intervals, effect sizes, degrees of freedom and *P* value noted<br>*Give P values as exact values whenever suitable.*
☒	☐	For Bayesian analysis, information on the choice of priors and Markov chain Monte Carlo settings
☒	☐	For hierarchical and complex designs, identification of the appropriate level for tests and full reporting of outcomes
☒	☐	Estimates of effect sizes (e.g. Cohen's *d*, Pearson's *r*), indicating how they were calculated

Our web collection on statistics for biologists contains articles on many of the points above.

Software and code

Policy information about availability of computer code

Data collection	Protein gel and immunoblot images were collected using BioRad Image Lab Touch 3.0.1.14.<br>ITC measurements were collected using MicroCal ITC200 Control Software 1.26 (GE).<br>AUC measurements were collected using ProteomeLab 6.0 (Beckman-Coulter).<br>Mass spectrometry data was collected in XCalibur 4.1.50 (Thermo Scientific).<br>Protein homologs were identified using the HHPred webserver.<br>Phylogenetic trees were constructed using the iTOL web server v6.<br>Predicted structure of Vs.4 that was created using AlphaFold Monomer v2<br>Luminescence and fluorescence measurements were collected using CLARIOstar software 5.61
Data analysis	PHENIX 1.17.1-3660-000, Coot 0.8.9.2, PHASER 2.8.3, PyMOL 2.3.2, HKL-3000, Image Lab 6.0, NITPIC 2.0.4, GUSSI 1.4.2, Proteome Discoverer 2.4.1.15 (Thermo Scientific), SEDFIT 16.1c, and SEDPHAT 14.0, WebLogo 3, GraphPad Prism 9.5.0, DNASTAR SeqMan NGen 17.3.0.59

For manuscripts utilizing custom algorithms or software that are central to the research but not yet described in published literature, software must be made available to editors and reviewers. We strongly encourage code deposition in a community repository (e.g. GitHub). See the Nature Portfolio guidelines for submitting code & software for further information.

March 2021

Data

Policy information about availability of data

All manuscripts must include a data availability statement. This statement should provide the following information, where applicable:

- Accession codes, unique identifiers, or web links for publicly available datasets
- A description of any restrictions on data availability
- For clinical datasets or third party data, please ensure that the statement adheres to our policy

The structure of Vs.4 from T4 phage in complex with cGAMP is publicly available at the RCSB Protein Data Bank (PDB ID: 7UQ2). E. coli strain K12 proteome database that was used in this study can be accessed at Uniprot (UP000000625). The T4 phage proteome database that was used in this study can be accessed at Uniprot (UP000009087). Vs.4 homologs can be accessed at the PHROGs database (PHROG 717). Sequence Read Archive (SRA) under BioProject PRJNA931786. GenBank accessions for the CBASS operons used in this study are found in the Methods. Materials including strains and plasmids are available upon reasonable request. Source data are provided with this paper.

Field-specific reporting

Please select the one below that is the best fit for your research. If you are not sure, read the appropriate sections before making your selection.

☒ Life sciences ☐ Behavioural & social sciences ☐ Ecological, evolutionary & environmental sciences

For a reference copy of the document with all sections, see nature.com/documents/nr-reporting-summary-flat.pdf

Life sciences study design

All studies must disclose on these points even when the disclosure is negative.

Sample size	Sample sizes were chosen to reliably determine the differences between groups. Given the large effects, we performed experiments in triplicate or quadruplicate to demonstrate reproducibility.
Data exclusions	No data were excluded from the analysis
Replication	All experimental findings that support the reported conclusions were repeated at least twice. All replication attempts were successful.<br><br>Exploratory experiments in this study were generally preformed once because their purpose was candidate identification. Candidates were verified in subsequent experiments. This includes the exploratory mass spectroscopy shown in Fig.1c, the IP-MS of cGAS conjugated proteins in T4 infected cells, and the forward genetic screen of T4 phage. The IP-MS of cGAS conjugated proteins was successfully replicated in two experiments.
Randomization	No experimental or control groups were randomly chosen because experiments were preformed in isogenic strains and there were no covariates to control for. A random set of R-free reflections was used in the refinement of the crystal structure
Blinding	Blinding was not necessary for this study because the data had strong, reproducible effects and the raw data are reported in the manuscript

Reporting for specific materials, systems and methods

We require information from authors about some types of materials, experimental systems and methods used in many studies. Here, indicate whether each material, system or method listed is relevant to your study. If you are not sure if a list item applies to your research, read the appropriate section before selecting a response.

Materials & experimental systems

n/a	Involved in the study	
☐	☒	Antibodies
☐	☒	Eukaryotic cell lines
☒	☐	Palaeontology and archaeology
☒	☐	Animals and other organisms
☒	☐	Human research participants
☒	☐	Clinical data
☒	☐	Dual use research of concern

Methods

n/a	Involved in the study	
☒	☐	ChIP-seq
☒	☐	Flow cytometry
☒	☐	MRI-based neuroimaging

Antibodies

Antibodies used	Monoclonal ANTI-FLAG M2 antibody produced in mouse(F1804, Sigma), Anti-mouse IgG, HRP-linked Antibody (7076, Cell Signaling),

Antibodies used	Anti-DYKDDDDK Magnetic Agarose (A36797, Pierce)
Validation	All the antibodies have been verified by the manufacturers according to their websites. The product information sheet for the primary antibody (ANTI-FLAG M2,F1804, Sigma) used in this study states that [this] "monoclonal antibody detects only the target protein band(s) on a Western blot from an E. coli, plant or mammalian crude cell lysate"

Eukaryotic cell lines

Policy information about cell lines

Cell line source(s)	THP1-Lucia ISG cells were obtained from InvivoGen (Cat. thpl-isg)
Authentication	This cell line was authenticated using morphology and functional assay (expression of a secreted luciferase (Lucia) reporter gene under the control of an IRF-inducible promoter).
Mycoplasma contamination	the cell line was not tested for mycoplasma contamination
Commonly misidentified lines (See ICLAC register)	No commonly misidentified lines were used in this study.
